# Recall Responses to Tetanus and Diphtheria Vaccination Are Frequently Insufficient in Elderly Persons

**DOI:** 10.1371/journal.pone.0082967

**Published:** 2013-12-11

**Authors:** Birgit Weinberger, Michael Schirmer, Raffaella Matteucci Gothe, Uwe Siebert, Dietmar Fuchs, Beatrix Grubeck-Loebenstein

**Affiliations:** 1 Institute for Biomedical Aging Research, University of Innsbruck, Innsbruck, Austria; 2 Department of Internal Medicine VI, Medical University Innsbruck, Innsbruck, Austria; 3 Department of Public Health and Health Technology Assessment, UMIT – University for Health Sciences, Medical Informatics and Technology, Hall in Tirol, Austria; 4 Institute for Technology Assessment and Department of Radiology, Massachusetts General Hospital, Harvard Medical School and Department of Health Policy and Management, Harvard School of Public Health, Boston, Massachusetts, United States of America; 5 Division of Biological Chemistry, Biocenter, Medical University, Innsbruck, Austria; Public Health England, United Kingdom

## Abstract

Demographic changes and a more active life-style in older age have contributed to an increasing public awareness of the need for lifelong vaccination. Currently many older persons have been vaccinated against selected pathogens during childhood but lack regular booster immunizations. The impact of regular vaccinations when started late in life was analyzed in an open, explorative trial by evaluating the immune response against tetanus and diphtheria in healthy older individuals. 252 persons aged above 60 years received a booster vaccination against tetanus, diphtheria, pertussis and polio and a subcohort (n=87) was recruited to receive a second booster vaccination against tetanus, diphtheria and pertussis 5 years later. The percentage of unprotected individuals at the time of enrollment differed substantially for tetanus (12%) and diphtheria (65%). Despite protective antibody concentrations 4 weeks after the first vaccination in almost all vaccinees, antibodies had again dropped below protective levels in 10% (tetanus) and 45% (diphtheria) of the cohort after 5 years. Protection was restored in almost all vaccinees after the second vaccination. No correlation between tetanus- and diphtheria-specific responses was observed, and antibody concentrations were not associated with age-related changes in the T cell repertoire, inflammatory parameters, or CMV-seropositivity suggesting that there was no general biological “non-responder type.” Post-vaccination antibody concentrations depended on pre-existing plasma cells and B cell memory as indicated by a strong positive relationship between post-vaccination antibodies and pre-vaccination antibodies as well as antibody-secreting cells. In contrast, antigen-specific T cell responses were not or only weakly associated with antibody concentrations. In conclusion, our findings demonstrate that single shot vaccinations against tetanus and/or diphtheria do not lead to long-lasting immunity in many elderly persons despite administration at relatively short intervals. Sufficient antigen-specific B cell memory B generated by adequate priming and consecutive booster vaccinations and/or exposure is a prerequisite for long-term protection.

**Trial Registration:**

EU Clinical Trials Register (EU-CTR); EudraCT number 2009-011742-26; www.clinicaltrialsregister.eu/ctr-search/trial/2009-011742-26/AT

## Introduction

Between 1990 and 2010, the percentage of persons aged 65 years or over has risen from 13.9% to 17.4% in the European population (EU-27) and is estimated to reach 30% by 2060 (European Commission, Demography Report 2010. http://ec.europa.eu/eurostat). The topic of vaccination in old age has received increasing attention in the last years resulting in new vaccination recommendations for the elderly in many countries [1,2]. Older persons are enjoying an increasingly active lifestyle leading to changes in their medical needs including their awareness of the importance of vaccination. Vaccines against influenza, pneumococcal disease or herpes zoster are not only an effective measure to prevent severe disease and mortality, but can also be a measure to ensure quality of life and independence. Presently, consensus is arising that regular vaccinations over the whole lifetime would be optimal [1,3–5]. This concept is still far away from reality, in particular for the generation over 60 years of age. Vaccination against tetanus has been available since the 1920s and most older persons have been vaccinated against tetanus during childhood. However, it has been shown that the number of vaccine doses received in life decreases with age. In a study conducted in France adults under the age of 30 years were shown to have received on average 7.1 (95%CI 6.9-7.2) doses of tetanus vaccine, which corresponds well with recommendations of 5 doses during childhood/adolescence and 10-year booster intervals in many countries. However, persons aged 50 to 60 years received only 5.7 (95%CI 4.6-6.8) during their lifetime indicating that booster vaccination was not regularly performed [6]. Whereas neonatal tetanus has virtually disappeared in Europe (0-7 cases per year from 2007-2011 compared to 69 and 27 in 1990 and 2000, respectively), there are still 100-200 cases of tetanus infection reported in Europe per year, mainly in adults over the age of 50 years (data from 2007-2011) [7]. Similar data have been obtained for the usage of diphtheria vaccine in persons up to 60 years of age in France with slightly lower numbers of vaccines doses during life-time compared to young adults [6]. However, recommendations regarding vaccination against diphtheria varied greatly in the last century and vaccination was presumably not performed during and shortly after World War II. Therefore persons born in the 1940s might frequently not have received appropriate childhood vaccination. Vaccination recommendations differ between European countries, but there is consensus that tetanus and diphtheria vaccination should be applied using a combined vaccine. In several countries pertussis is included as an additional antigen in combination vaccines. In many cases, vaccination history is better documented for tetanus than for diphtheria, and the time point for booster vaccination is based on the last tetanus vaccination. In the summary of product characteristics of combination vaccines containing tetanus and diphtheria toxoid administration of three doses in a primary schedule is described for persons in whom the last vaccination dates back more than 20 years. However, in clinical practice this is rarely done. We and others have demonstrated decreased antibody concentration and lack of protection against tetanus and diphtheria in old age [8–12]. We also demonstrated that protection was relatively short lasting in old age [13]. The goal of this study was therefore to investigate the level of protection against tetanus and diphtheria in the elderly population and to analyze the immune response to tetanus and diphtheria following two doses of vaccine applied at a 5-years interval. A cohort of 252 persons aged 60 years or older received a booster vaccination against tetanus, diphtheria, pertussis and polio. The results of this study have been published previously [14]. For the current study 87 persons from this cohort were recruited to receive a second booster vaccination five years later. In accordance with national vaccination guidelines the second booster vaccination did not include polio antigen. As vaccination history is very heterogeneous for pertussis and natural exposure is more likely for this pathogen we chose to investigate only tetanus- and diphtheria-specific immune responses. Thereby we aimed to evaluate the impact of regular vaccinations in old age in a “real-life” cohort with presumably frequently inadequate vaccination history. We demonstrate that the chosen immunization strategy does not lead to long lasting immunity in many elderly persons due to a too small memory B cell/plasma cell pool.

## Materials and Methods

### Study cohort

The protocol for this trial and a supporting CONSORT checklist are available as supporting information; see Protocol S1 and Checklist S1. 

**Figure 1:**
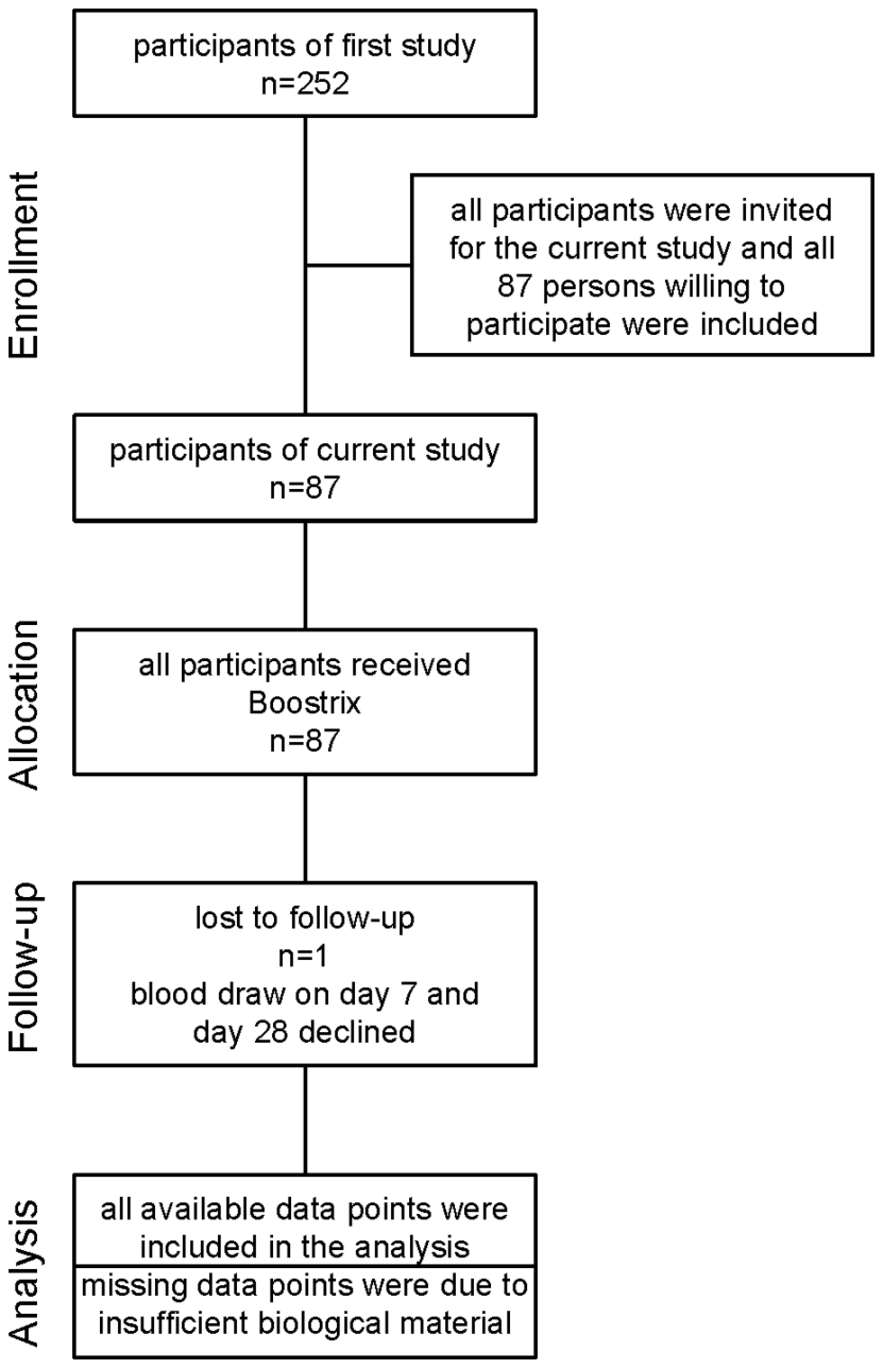
CONSORT flow diagram of the study.

The original study cohort included 252 healthy, elderly volunteers (median age 66y, range 59-91y; 116 females) who received a booster vaccination against tetanus, diphtheria, pertussis and polio (Repevax^®^, sanofi pasteur MSD). Antibody concentrations against all components of the vaccine were determined prior to and 4 weeks after vaccination and the results have been previously published [14]. For the current study a subcohort of 87 persons received a second booster vaccination (Boostrix® 0.5ml, Glaxo Smith Kline) 5 years later (recruitment: January 2010-April 2010; follow-up: 4 weeks after vaccination). In accordance with Austrian vaccine recommendations [15], the second vaccination did not include polio antigens. Both vaccines are commercially available and contain aluminum phosphate and aluminum hydroxide as an adjuvant. Figure 1 shows the CONSORT flow chart for the study. For the current study report serum samples obtained before and after the first vaccination were re-tested for the 87 persons receiving the second vaccination in order to ensure comparability of the methods. The antibody concentrations obtained in the re-testing corresponded well with the original data (Spearman correlation: tetanus: r_s_=0.878. and r_s_=0.841. pre- and post-vaccination; diphtheria: r_s_=0.895 and r_s_=0.905 pre- and post-vaccination; p<0.0001 for all correlations). Self-reported vaccination history at the time of the first study enrollment was heterogeneous and is summarized in Table 1. Persons with chronic viral infection (Human Immunodeficiency virus, Hepatitis B virus, Hepatitis C virus), transplant recipients and patients under immunosuppressive or chemotherapy were not included in the study. Routine laboratory parameters (liver and kidney function, blood count) were determined and all participants were shown to be in good health. No serious adverse events occurred after vaccination. The primary objective of the study was to compare vaccine-induced immune responses in young versus elderly adults. This comparison is subject of a separate publication currently in preparation. The results presented here cover the secondary objective of the study, which was to identify factors influencing vaccination outcome.

**Table 1:** Self-reported vaccination history at the time of enrollment.

Tetanus	time since last vaccination known	n=78	10.1 ± 5.2 years
	time since last vaccination unknown	n=9	-
Diphtheria	time since last vaccination known	n=41	15.1 ±17.1 years
	time since last vaccination unknown	n=46	-
Tetanus/Diphtheria	simultaneously	n=27 (31%)	

The number of participants for whom the time point of the last vaccination was known or unknown known is indicated. The mean time ± SD since the last vaccination is given in years. The number and percentage of persons, who received their last vaccination against tetanus and diphtheria as a combined vaccine, are indicated.

### Ethics statement

Both studies were approved by the local ethics committee (Medical University, Innsbruck, Austria) and in accordance with changes in the legal requirements the second vaccination was registered at the EU Clinical Trials Register (EU-CTR) as an open exploratory Phase 4 clinical trial with the EUDRACT number 2009-011742-26. All participants gave their written informed consent.

### Determination of antibody concentrations

Microtiter plates were coated with 1µg/ml tetanus or diphtheria toxoid (Statens Serum Institute) and blocked with 0.01M Glycin. Serum samples were tested in duplicates. Peroxidase-labeled rabbit anti-human IgG (Chemicon/Millipore) antibody was used as secondary antibody. IgG antibodies were quantified in IU/ml using standard human anti-tetanus and anti-diphtheria sera (NIBSC). The detection limit of the assays used was 0.01 IU/ml and values below the limit of detection were set to 0.005 IU/ml for calculation of geometric mean concentrations (GMC). Antibody concentrations above 0.1 IU/ml were considered as protective.

Antibodies against Cytomegalovirus (CMV) were determined using a commercially available ELISA Kit (Siemens).

### Detection of antigen-specific antibody secreting cells

ELISPOT assays for detection of antigen-specific antibody-secreting cells were performed following manufacturer´s instructions (Mabtech). Briefly, PVDF-membrane 96-well plates (MAIPS4510, Millipore) were pre-wetted with 70% ethanol and coated over night with 10µg/ml anti-IgG antibody (Mabtech). 500.000 peripheral blood mononuclear cells (PBMC) per well were cultivated for 24h. Detection of antigen-specific antibody-producing cells was performed using 0.05µg/ml tetanus or diphtheria toxoid (1.5h, 37°C), which had been biotinylated using the EZ-Link Sulfo-NHS-LC-Biotinylation Kit (Pierce). Detection was performed using Streptavidin coupled with alkaline phosphatase (1.5h, RT) and the colorimetric substrate BCIP/NBT (Moss Inc.). Spots were counted using a Zeiss Elispot Reader. Detection with biotinylated bovine serum albumin was used as a negative control.

### Detection of antigen-specific cytokine secreting T cells

ELISPOT assays for detection of antigen-specific T cells secreting Interferon (IFN)-γ or Interleukin (IL)5 were performed following manufacturer´s instructions (Mabtech). Briefly, PVDF-membrane 96-well plates (MAIPS4510, Millipore) were pre-wetted with 70% ethanol and coated over night with 5µg/ml anti-IFN-γ or anti-IL-5 antibody (Mabtech). 500.000 PBMC per well were cultivated for 24h (IFN-γ) or 48h (IL-5) in the presence of 5µg/ml tetanus or diphtheria toxoid. Detection of secreted cytokines was performed by adding 1µg/ml biotinylated anti-INF-γ or anti-IL-5 antibody (Mabtech, 1.5h, 37°C). Detection was performed using Streptavidin coupled with alkaline phosphatase (1.5h, RT) and the colorimetric substrate BCIP/NBT (Moss Inc.). Spots were counted using a Zeiss Elispot Reader. PBMC cultured without antigen served as a negative control.

### Flow cytometry

PBMC were washed with PBS and stained with anti-CD3-PE-Cy7 (Biolegend), anti-CD4-PerCP (BD Pharmingen), anti-CD8-PE (BD Pharmingen), anti CD28-APC (Biolegend),and anti CD45RO-(FITC (BD Pharmingen) antibodies for 20 min, 4°C in the dark. After washing with PBS, cells were analyzed using a FACS Canto II cytometer and FACSDiva software (BD). T cells were gated as CD3^+^CD4^+^ or CD3^+^CD8^+^ and naïve (CD28^+^CD45RO^-^), memory (CD28^+^CD45RO^+^) and effector (CD28^-^) subpopulations were defined.

### Quantification of IL-6 and IL-10 in serum

Serum concentrations of Interleukin (IL)-6 and IL-10 were determined by commercially available ELISA Kits (Mabtech) following manufacturer´s instructions.

### Determination of neopterin, kynurenine, tryptophan and CRP

Tryptophan and kynurenine concentrations were determined by HPLC on the same day using a reversed-phase C_18_ column, 0.015 mol/l acetic acid/sodium acetate buffer and a flow-rate of 1 ml/min as described earlier [16,17]. Tryptophan was monitored by its native fluorescence at 285 nm excitation and 360 nm emission wavelengths, kynurenine was determined by UV absorption detection at 360 nm wavelength, and the kynurenine to tryptophan ratio (Kyn/Trp) was calculated as an estimate of the tryptophan breakdown rate. Serum neopterin concentrations were measured using a competitive ELISA with a sensitivity of 2 nmol/l (Brahms). The concentration of C-reactive protein (CRP) in serum was determined by a diagnostic laboratory using a turbidimetric method.

### Statistical analysis

Continuous variables are expressed as median and range as well as means ± standard deviation. The Spearman’s rank correlation coefficient was calculated to determine relationships between tetanus- and diphtheria-specific antibody concentrations and other baseline characteristics prior to vaccination with concentrations after vaccination and changes in concentration during follow-up. Scatterplots were used to show the relationship patterns. Correlations between tetanus- and diphtheria-specific responses were also analyzed using Spearman´s rank correlation coefficients. In order to explore general biological “non-responder phenotypes”, dependence of antibody concentrations with age-related changes in the T cell repertoire and inflammatory parameters were examined by Spearman´s rank correlation coefficients. Antibody responses in CMV-seropositive and CMV-seronegative persons were compared by Mann-Whitney U-test. A two-sided p-value of less than 0.01 was considered to indicate statistical significance. All statistical analyses were performed using the software package SPSS version 11 (SPSS Inc., Chicago, US). 

## Results

### Level of protection against tetanus and diphtheria in elderly vaccinees

**Figure 2:**
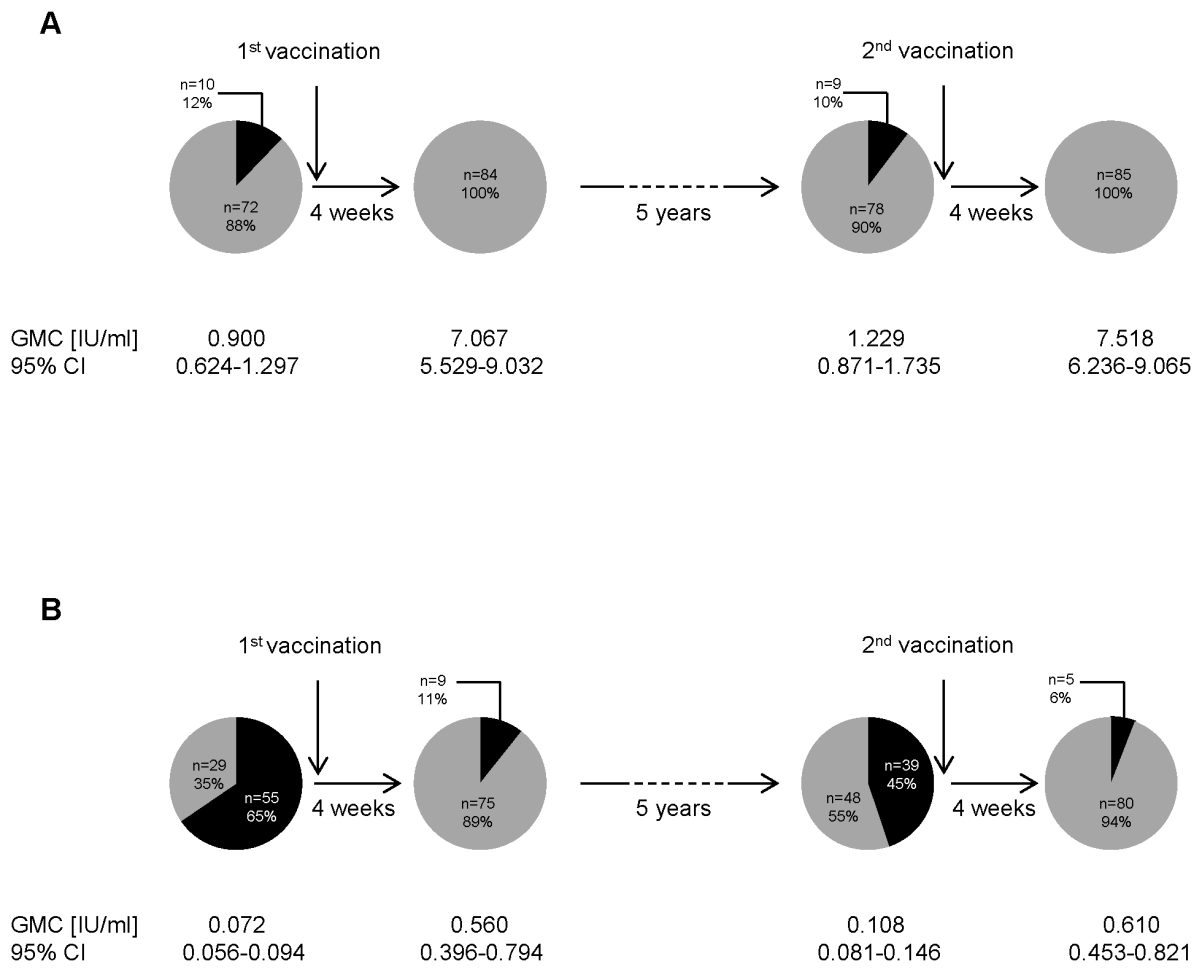
Percentage of vaccinees protected against tetanus and diphtheria and geometric mean antibody concentrations. The percentages of vaccinees with tetanus (A)- or diphtheria (B)-specific antibody concentrations above (grey) or below (black) 0.1 IU/ml, which is considered to be protective, are depicted in pie charts. Percentages and numbers of vaccinees are indicated. The geometric mean concentrations (GMC) and 95% confidence intervals (CI) are indicated below the pies. Antibody concentrations were measured by ELISA prior to and four weeks after the two consecutive vaccinations, which were administered at a 5-year interval.

Antibody concentrations were determined prior to and 28 days after each of two vaccinations, which were administered at a five-year interval. For tetanus we found that 12% of the cohort had antibody concentrations below 0.1 IU/ml prior to the first vaccination (Figure 2A). Four weeks after the vaccination all participants developed antibody concentrations above the protective limit of 0.1 IU/ml. Over the 5-year interval until the second vaccination antibody concentrations dropped under the protective limit in 10% of the participants and again all vaccinees developed antibody concentrations above 0.1IU/ml four weeks after the second vaccination. For diphtheria, our results demonstrated that 65% of the participants did not have protective antibody concentrations prior to the first vaccination (Figure 2B). The majority of these persons developed protective antibodies, but 11% of the vaccinees still had no protective antibody concentrations four weeks after the first vaccination. Over the 5-year interval (i.e., between the two vaccinations), antibody concentrations declined below protective levels in a large portion of the participants, leaving 45% without protective antibodies prior to the second vaccination. Four weeks after the second booster vaccination, 6% of the vaccinees still had no protective antibodies. These results demonstrate that the levels of protection against tetanus and diphtheria differ substantially in the older population. Despite the fact that even with very low or undetectable pre-vaccination antibody titers most older persons mount protective immune responses to tetanus and diphtheria after a single shot, the duration of the protection is short in a large proportion of the cohort, particularly in the case of diphtheria.

### Correlations of antibody concentrations after the first and second vaccination

**Figure 3:**
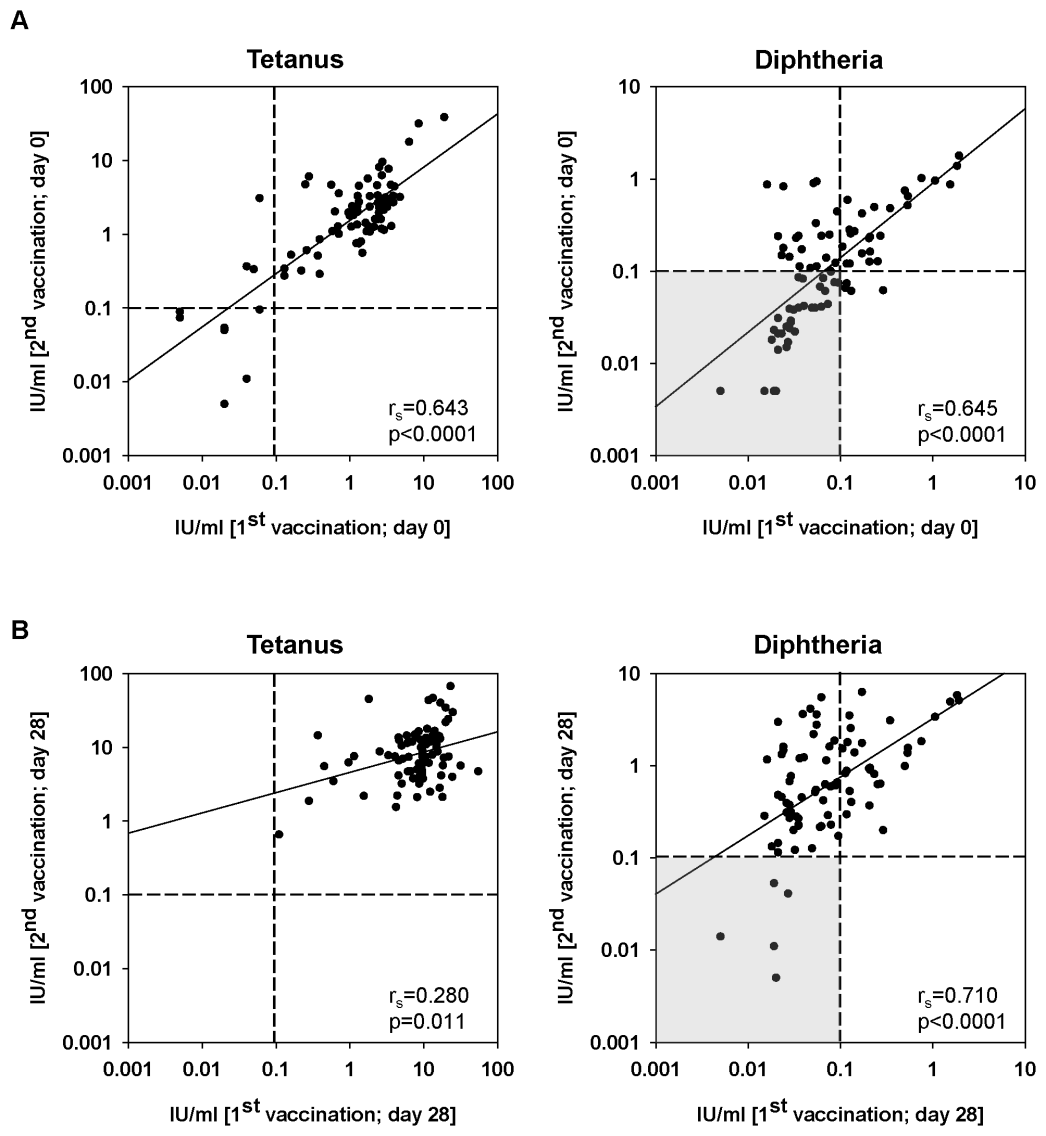
Correlation of antibody concentrations prior to and after vaccination. Correlations of pre- (A) and post- (B) antibody concentrations for the first and second vaccination are shown. The Spearman correlation coefficient (r_s_) and the p-value (Spearman's rank correlation) are depicted for each diagram. The grey insert highlights individuals without protective diphtheria-specific antibody levels for both doses of vaccine. p-values <0.05 are considered as significant.

We next wanted to define whether there were consistent responders/non-responders to tetanus- and/or diphtheria-vaccination. We first correlated antibody concentrations before the first with the ones before the second vaccination. Figure 3A shows that the antibody concentrations prior to the first shot were highly correlated with the antibody concentration prior to the second vaccination for both tetanus and diphtheria (p<0.0001). This relationship was also observed when antibody concentrations after the first and after the second vaccination were compared (p=0.011 for tetanus; p<0.0001 for diphtheria) (Figure 3B). This suggests a persistent high-responder/low-responder pattern. In the case of diphtheria 40% of the cohort did not have protective antibody concentrations, neither before the first nor before the second vaccination (indicated grey in Figure 3A). Five of these persons were additionally unable to raise a protective immune response against diphtheria, neither after the first nor the second vaccination (indicated grey in Figure 3B).

### Antibody concentrations to tetanus and diphtheria are not linked

In order to define whether there was a “non-responder phenotype” defined by a general inability of certain elderly persons to respond properly to vaccination, we correlated antibody concentrations to tetanus and diphtheria prior to and after both vaccinations.

**Figure 4:**
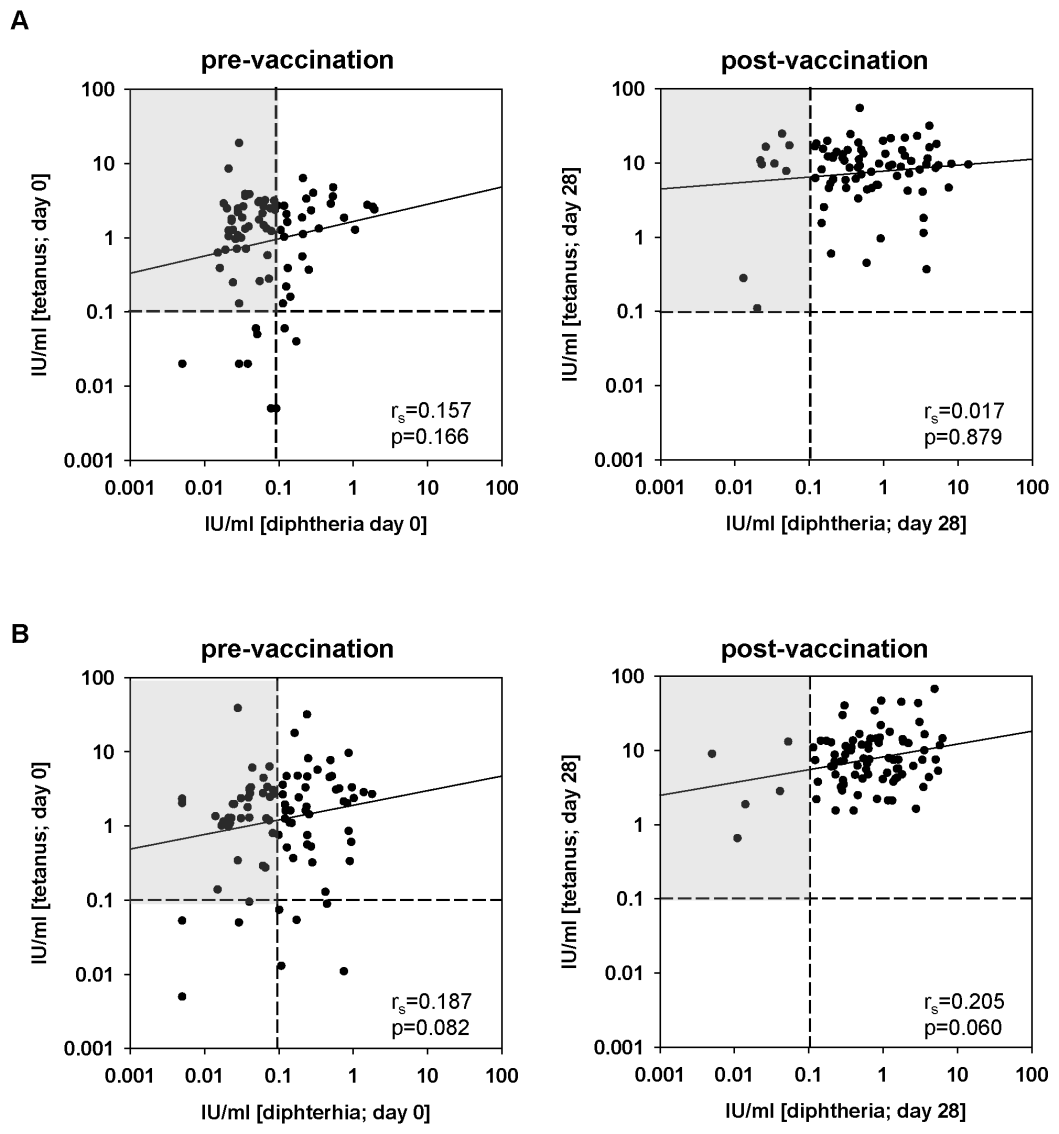
Correlations of tetanus- and diphtheria-specific antibodies prior to and after vaccination. Correlations of tetanus- and diphtheria-specific antibodies prior to and after the first (A) and the second (B) vaccination are shown. The Spearman correlation coefficient (r_s_) and the p-value (Spearman´s rank correlation) are depicted for each diagram. The grey insert highlights individuals with high tetanus-specific antibody concentrations despite low levels of diphtheria-specific antibodies. p-values <0.05 are considered as significant.

Figure 4 demonstrates that there was no statistically significant relationship between tetanus- and diphtheria-specific antibody concentrations before and after the first vaccination (p=0.166 pre-vaccination; p=0.879 post-vaccination). Correlations between tetanus- and diphtheria-specific antibodies are slightly more pronounced for the second vaccination suggesting a certain degree of synchronization, but still do not reach statistical significance (p=0.082 pre-vaccination; p=0.060 post-vaccination). These results show that there is no general non-responder type but that inability to respond seems to be linked to a certain antigen. Parameters used as indicators of biological aging of the immune system [18–25] did also not correlate with antibody concentrations before and after vaccination. Specifically we measured CD4^+^ and CD8^+^ naïve, memory and effector T cell counts, neopterin, the kynurenin/tryptophan ratio in serum, as well as parameters of inflammation (“inflammaging”) [26], such as IL-6, IL-10, and CRP (Table 2). In addition, there was no difference in antibody concentrations comparing CMV-seronegative and CMV-seropositive individuals (data not shown). Latent CMV infection is believed to accelerate the aging of the immune system [27,28].

**Table 2:** Correlations between immunological markers and antibody concentrations prior to and after the second vaccination.

TETANUS				
	antibodies	pre-vaccination	antibodies	post-vaccination
	r_s_	p-value	r_s_	p-value
CD4+ naive	-0.178	0.298	-0.080	0.644
CD4+ memory	0.156	0.364	0.036	0.835
CD4+ effector	-0.023	0.892	0.119	0.488
CD8+ naive	-0.042	0.807	0.053	0.760
CD8+ memory	-0.004	0.981	-0.069	0.688
CD8+ effector	0.052	0.762	0.072	0.674
IL-6	-0.042	0.701	0.009	0.932
IL-10	0.155	0.151	0.053	0.633
CRP	-0.266	0.014	-0.115	0.296
neopterin	-0.077	0.477	0.015	0.889
kynurenine/tryptophan	0.049	0.652	0.212	0.052
DIPHTHERIA				
	antibodies	pre-vaccination	antibodies	post-vaccination
	r_s_	p-value	r_s_	p-value
CD4+ naive	-0.141	0.413	-0.261	0.124
CD4+ memory	0.274	0.106	0.316	0.060
CD4+ effector	-0.349	0.037	-0.072	0.675
CD8+ naive	0.106	0.540	0.066	0.703
CD8+ memory	0.201	0.241	0.188	0.273
CD8+ effector	0.199	0.244	-0.234	0.169
IL-6	0.055	0.614	0.118	0.283
IL-10	0.117	0.280	0.193	0.076
CRP	-0.103	0.346	0.023	0.836
neopterin	-0.027	0.805	0.093	0.396
kynurenine/tryptophan	0.028	0.796	0.116	0.291

Correlations of immunological parameters and antibody concentrations before and after the second vaccination for tetanus and diphtheria. r_s_ = Spearman correlation coefficient; p-values ≤0.01 are considered significant.

### Antibody concentrations following vaccination depend on antigen-specific B but only modestly on T_h2_ cell memory

**Figure 5:**
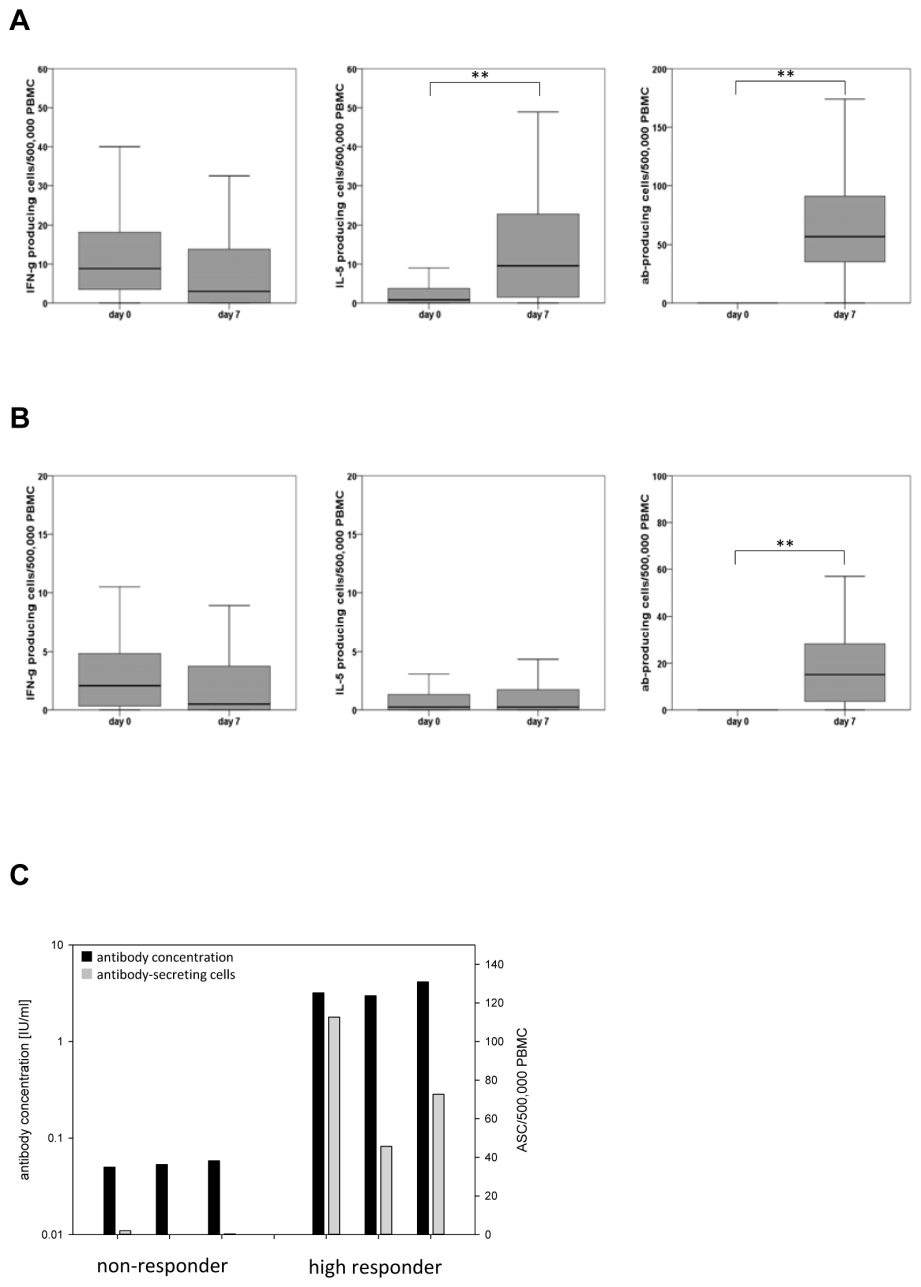
Antigen-specific cytokine secreting T-cells and antibody-secreting T cells. A Box plots of tetanus-specific IFN-γ- (left panel) and IL-5- producing (right panel) T cells and IgG-secreting B cells (right panel) as measured by ELISPOT before (day0) and 7 days after vaccination. Boxes represent the 25th and 75th percentile and medians are indicated. Whiskers indicate the 5th and 95th percentile. ** p<0.01 (Wilcoxon signed-rank test). B Box plots of diphtheria-specific IFN-γ- (left panel) and IL-5- producing (right panel) T cells and IgG-secreting B cells (right panel) as measured by ELISPOT before (day0) and 7 days after vaccination. Boxes represent the 25th and 75th percentile and medians are indicated. Whiskers indicate the 5th and 95th percentile. ** p<0.01 (Wilcoxon signed-rank test). C Representative examples for persons without (n=3) or with high (n=3) diphtheria-specific antibody concentrations following vaccination Depicted are antibody concentrations 28 days after vaccination (black) and diphtheria-specific antibody secreting cells (ASC) 7 days after vaccination (grey).

To define which part of the adaptive immune response was decisive for antibody concentrations after vaccination, we determined antigen-specific T cell responses as well as antibody secreting cells 7 days after vaccination. Generally, more tetanus- than diphtheria-specific T cells were detected prior to and after the vaccination and the number of IL-5 secreting diphtheria-specific T cells was generally low (Figure 5A & B). Associations between antibody concentrations on day 28 and T cell and B cell responses on day 7 were analyzed. While antigen-specific IFN- γ and IL-5 production did not correlate with antibody concentrations for tetanus (p=0.258 and p=0.065), there was a correlation between antibody concentrations and IL-5 (p=0.002), but not IFN-γ production (p=0.036) for diphtheria. In contrast, there was a strong positive relationship between the number of ASC and post-vaccination antibody concentrations for both tetanus and diphtheria (Table 3). 

**Table 3:** Correlation between antibody concentrations and cellular immune responses.

TETANUS			
		r_s_	p-value
antibodies d28	IFN-γ secreting T cells	0.124	0.258
	IL-5 secreting T cells	0.201	0.065
	antibody secreting cells	0.446	<0.0001
DIPHTHERIA			
		r_s_	p-value
antibodies d28	IFN-γ secreting T cells	0.228	0.036
	IL-5 secreting T cells	0.338	0.002
	antibody secreting cells	0.759	<0.0001

Correlations of antibody concentrations (day 28) with specific IFN-γ or IL-5 secreting T cells and with specific antibody secreting cells for tetanus and diphtheria. r_s_ = Spearman correlation coefficient; p-values ≤0.01 are considered significant.

The number of antibody secreting cells detected in the periphery 7 days after booster vaccination (Figure 5 A&B) reflects stimulation of memory B cells, which have been generated by previous immunizations. Persons repeatedly not responding to vaccination against diphtheria (highlighted in grey in Figure 3B, right panel) did characteristically not have detectable numbers of antibody secreting cells on day 7 (Figure 5C) suggesting that their memory B cell pool was too small to allow a protective booster response. Similar to our previous work [14] we saw a positive correlation of pre- and post-vaccination antibody concentrations (tetanus: r_s_=0.311, p=0.005; diphtheria: r_s_=0.668, p≤0.0001 for the first vaccination and tetanus: r_s_=0.404, p≤0.0001; diphtheria: r_s_=0.708, p≤0.0001 for the second vaccination). In summary, these results demonstrate that antibody responses following vaccination against tetanus and diphtheria greatly depend on B cell memory and pre-existing plasma cells but only to a low extent on T_h2_ and not on T_h1_ T cell memory in elderly persons.

## Discussion

We investigated the level of protection against tetanus and diphtheria in the elderly population and analyzed the immune response to tetanus and diphtheria following two doses of vaccine applied at a 5-years interval. We were able to demonstrate that the levels of protection against tetanus and diphtheria differ substantially in the elderly population. 

Vaccination history was highly discordant for tetanus and diphtheria with only 31% of the participants having received their last vaccinations against both antigens simultaneously. 64% of the cohort had been vaccinated against tetanus in the last 10 years. In contrast, only 33% of the cohort had received vaccination against diphtheria in the last 10 years and 50% of the participants did not know whether they had ever been vaccinated against diphtheria. After a single booster shot of combined tetanus/diphtheria vaccine most individuals developed protective antibody concentrations against tetanus and diphtheria. However, 11% of our vaccinees did not have protective antibodies against diphtheria 4 weeks after vaccination. After 5 years 10% of the cohort had tetanus-specific antibodies below the threshold considered to be protective and almost half of the participants had already lost protective diphtheria-specific antibody concentrations. These had mostly also been unprotected at the time of enrollment (see Figure 3A). It has previously been shown that adolescents receiving one booster dose of tetanus and diphtheria vaccine are fully protected after 5 years, but that antibody concentrations decrease over time in adults under the age of 60 years leaving 40-50% unprotected against diphtheria after 5 years [29]. Taking into account that half of our cohort did not have a documented history of diphtheria vaccination it seems possible that they were never vaccinated against diphtheria and that the single shot applied with the low dose diphtheria component vaccine (dT) represents insufficient primary immunization. The fact that a relatively high number of persons lost protective immunity within 5 years supports this explanation. It is not yet known how long the effect of the second vaccination will last, but studies are on the way in our laboratory clarifying this issue. It has got to be kept in mind that a relatively large number of persons within the study cohort were unprotected for at least part of the 5-year observation period and therefore vulnerable to disease in the case of exposure.

Our study protocol followed the procedures likely being utilized in general practice, that is, the application of a single shot of combined vaccines containing tetanus and a reduced dose of diphtheria after assessment of the vaccination status for tetanus, which was relatively well documented for the majority of the cohort. Our data demonstrated that this approach most likely leaves large parts of the elderly population unprotected against diphtheria. Lack of protection against diphtheria may also represent a problem in respect to other vaccinations, as diphtheria toxoid or derivatives thereof are frequently used in conjugate vaccines. The relatively low efficacy of pneumococcal conjugate vaccines in elderly persons may thus be partly explained by a lack of carrier-specific immune responses [30,31].

The assumption that state-of-the-art primary immunization was missing is also supported by the fact that no ASC were found in the persons with the lowest antibody concentrations and the very good correlation between ASC and antibody responses. Our cohort thus partly seemed to have a too small diphtheria-specific memory B cell pool and T cell help was only borderline detectable in the form of low IL-5 production during the response. In contrast there were sufficiently high numbers of tetanus-specific ASC, and T cell help was obviously not necessary to activate them. It has been shown that age-related changes of the T cell compartment, interpreted as indicators of biological aging of the immune system, are correlated with impaired immune responses to influenza vaccination [19,32,33] and that the antibody response to pneumococcal vaccination is decreased in frail elderly [34], which show increased levels of inflammatory molecules, such as IL-6 and neopterin suggestive of “inflammaging” [26,35,36]. However, in our study tetanus- and diphtheria-specific antibody concentrations were independent of inflammatory markers and the composition of the T cell compartment. Due to the limited sample size and the multiple testing when investigating the dependence of concentrations on other characteristics the results should still be interpreted with caution.

In addition, we did not observe a significant correlation between tetanus- and diphtheria-specific immune responses. We therefore conclude that there is presumably not a general “non-responder” type possibly due to the biological aging process, but that responses to booster vaccination depend on pre-existing plasma cells as indicated by antibody concentrations prior to vaccination and B cell memory as indicated by specific ASC 7 days post vaccination. 

The proportion of persons protected against tetanus was higher in our present cohort than in previous ones [10,13], which may be explained by the fact that the participants of the present study were recruited at the Public Health Department of the Federal State of Tyrol, where they went for regular visits. This indicates that they were quite health conscious and aware of the necessity to be vaccinated at least against tetanus. However, vaccination coverage and protection was low for diphtheria even in this population. Discordant levels of protection against tetanus and diphtheria were also demonstrated in a serological survey in the UK, which showed that 36% or 72% of a cohort older than 70 years had antibody concentrations below 0.1IU/ml for tetanus and diphtheria, respectively [37]. This emphasizes that public awareness among doctors as well as patients regarding the necessity of vaccination can be present for one vaccine but not for others

The discrepancy between the immunization situation for tetanus and diphtheria in elderly persons will have to be addressed by future vaccination strategies. To achieve optimal immunization for both antigens different approaches are imaginable: Firstly, the tetanus component could be applied separately as in the past. This would have the disadvantage that the rate of diphtheria vaccination would presumably still drop unless public awareness of the necessity of diphtheria vaccination was raised. The advantage of this approach would be that proper primary immunization regimes against diphtheria could be performed. The success of primary immunization relatively late in life remains still to be elucidated, as the problem of memory generation late in life is well documented in animal models [38,39]. An alternative approach might be the use of different adjuvants for the three component vaccine. Substances such as MF59 have been shown to work well in seasonal as well as pandemic influenza vaccines [40–42] and it might therefore be possible to combine a sufficient booster effect for tetanus with the induction of a good primary response for diphtheria.

Taken together our results demonstrate that even vaccines as well known and common as the combined tetanus and diphtheria vaccine may not give satisfactory protection against all components in the elderly population. This represents a severe public health problem, which will have to be addressed in the coming years.

## Supporting Information

Checklist S1 CONSORT checklist.
(TIF)

Protocol S1 Trial protocol (english).
(PDF)

Protocol S2 Trial protocol (german).
(PDF)
