# Supplementary figures and images for: Recall Responses to Tetanus and Diphtheria Vaccination Are Frequently Insufficient in Elderly Persons
Source: PLoS One. 2013 Dec 11;8(12):e82967. doi: 10.1371/journal.pone.0082967 (PMC3859625; doi:10.1371/journal.pone.0082967)

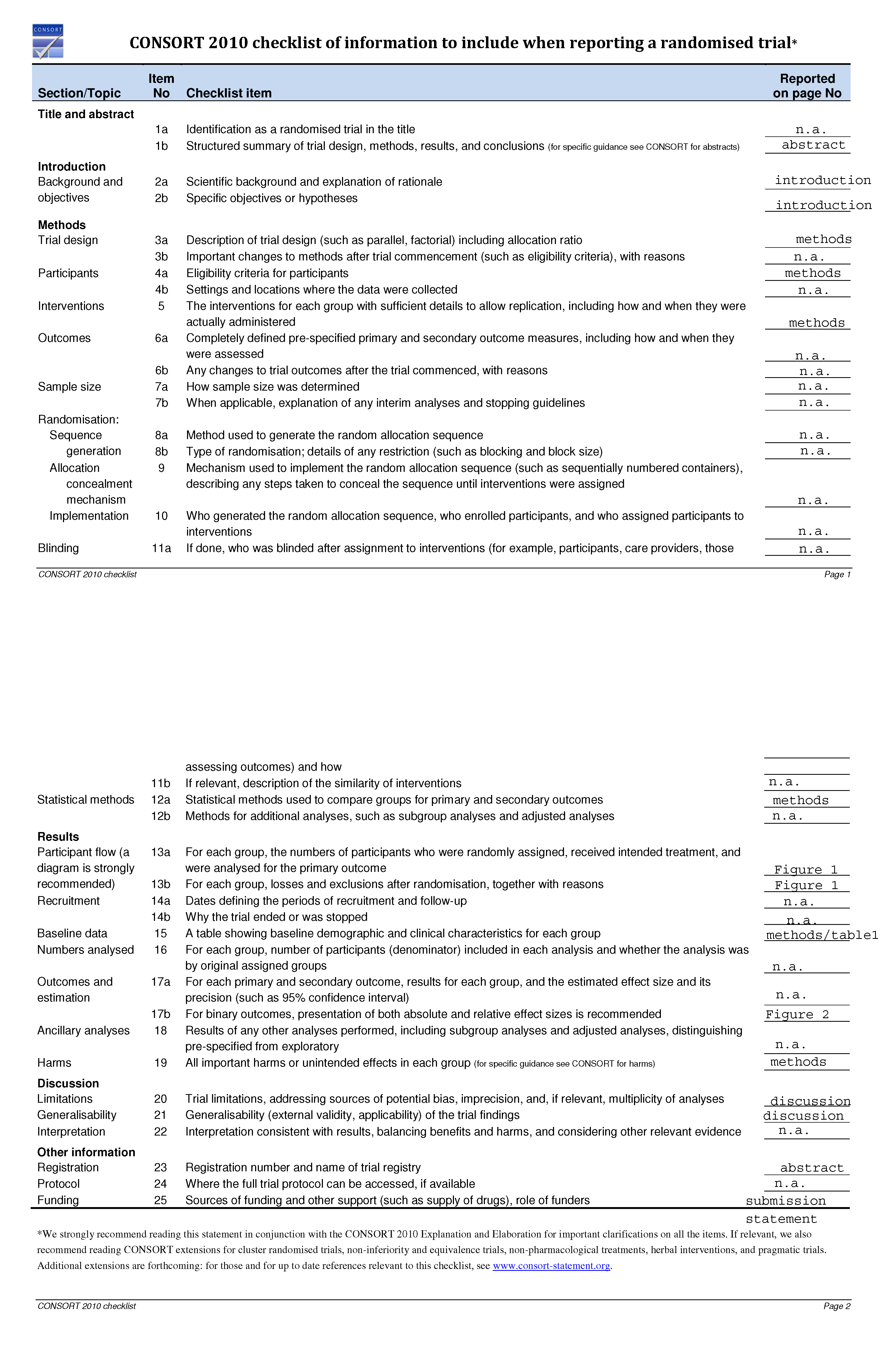

Supplement: Checklist S1 — CONSORT checklist. (TIF) [file pone.0082967.s001.tif]
