# Supplementary material for: Recall Responses to Tetanus and Diphtheria Vaccination Are Frequently Insufficient in Elderly Persons
Source: PLoS One. 2013 Dec 11;8(12):e82967. doi: 10.1371/journal.pone.0082967 (PMC3859625; doi:10.1371/journal.pone.0082967)
Supplement: Protocol S1 — Trial protocol (english). (PDF) [file pone.0082967.s002.pdf]

Trial Protocol

Version 2, 23.03.2009

Confidential

Title	Humoral and cellular immune response after booster vaccination against tetanus and diphtheria
Short title	Immune response after tetanus/diphtheria booster
Sponsor	Institute for Biomedical Aging Research<br>Rennweg 10<br>6020 Innsbruck<br>Austria
Sponsor´s protocol code	TetDip-2009
EudraCT-number	2009-011742-26

SPONSOR

Institute for Biomedical Aging Research
Rennweg 10
6020 Innsbruck
Austria

Confidentiality

All information provided in this trial protocol is strictly confidential and are provided to participating and potential physicians, health authorities and ethics committees for inspection and evaluation. Publication or distribution is not permitted without prior written consent of the sponsor. This does not apply to the “informed consent forms”, which may be used to obtain consent of potential participants.

All provisions of this protocol are binding for all signing parties.

Declaration of the sponsor

This study protocol has been carefully evaluated. Its content is in accordance with the current benefit/risk assessment for the methods described as well as with the moral, ethical and scientific principles of Good Clinical Practice, the last version of the Declaration of Helsinki and the local laws and regulations.

**Prof. Beatrix Grubeck-Loebenstein**<br>*Representative of the Sponsor (Institute for Biomedical Aging Research) and Principal Investigator*	Innsbruck, 26.03.09<br>City, date, signature

The signing parties declare, that they have read the trial protocol and that the trial protocol contains all relevant information needed to conduct the study. They further declare that they will conduct the trial in accordance with the current protocol. It is agreed that all not yet public information is strictly confidential.

SIGNITURES

Substance to be tested	**Boostrix and Boostrix Polio**
Title	Humoral and cellular immune response after booster vaccination against tetanus and diphtheria
Short title	Immune response after tetanus/diphtheria booster
EUDRACT number:	2009-011742-26

Declaration of the biometrician, the study coordinator and the responsible monitor:

I read the protocol and agree with its content. I declare, that it contains all information necessary for the conduct of the study. I agree to conduct the trial according to the current protocol. In particular, I will abide by the moral, ethical and scientific principles of Good Clinical Practice (GCP), the last version of the Declaration of Helsinki, local laws and regulations and relevant regulatory requirements.

Alterations of the protocol, which lie in the responsibility of any signing person will be declared immediately.

I agree to keep all information and documentation, which is not yet published, strictly confidential. This agreement includes the trial protocol, case report forms and other scientific data.

**Prof. Uwe Siebert**<br>*Biometrician*	Hall, 26.03.09<br>City, date, signature
**Dr. Birgit Weinberger**<br>Study coordinator	Innsbruck, 26.03.09<br>City, date, signature
**KKS Innsbruck**<br>*Monitor*	Innsbruck, 26.03.09<br>City, date, signature

Personnel / Institutions

Sponsor:

Institute for Biomedical Aging Research, represented by
Prof. Beatrix Grubeck-Loebenstein
Rennweg 10
6020 Innsbruck
Tel: 0512/583919-10
Fax: 0512/583919-8
beatrix.grubeck@oeaw.ac.at

Laboratory:

Dr. Birgit Weinberger
Rennweg 10
6020 Innsbruck
Tel: 0512/583919-13
Fax: 0512/583919-8
birgit.weinberger@oeaw.ac.at

Laboratory Dr. Philadelphy
Andreas-Hofer-Str. 28
6020 Innsbruck
Tel:0512/58021
Fax: 0512/58021-20
labor.philadelphy@phillab.at

Vaccination:

Landessanitätsdirektion für Tirol (Public Health Department, Federal State of Tyrol)
Dr. Christoph Neuner
Eduard-Wallnöfer-Platz 3
6020 Innsbruck
Tel: 0512/508-2662
Fax: 0512/508-2665
sanitaetsdirektion@tirol.gv.at

Study coordinator:

Dr. Birgit Weinberger

Data management:

Dr. Birgit Weinberger

<u>Monitoring:</u>

Koordinierungszentrum für Klinische Studien (KKS)
Coordinating center for clincial trials
Anichstr. 35
6020 Innsbruck
Tel: 0512/900370086
Fax: 0512/900370086
ctc@i-med.ca.at

<u>Biometric analysis:</u>

Prof. Uwe Siebert
UMIT
Department of Public Health, Medical Decision Making and Health Technology Assessment
Eduard-Wallnöfer-Zentrum 1
6060 Hall
Tel: 050/8648-3939
Fax: 050/8648-673930
public-health@umit.at

Synopsis

**Sponsor**	Institute for Biomedical Aging Research
**Title**	Humoral and cellular immune response after booster vaccination against tetanus and diphtheria
**Short title**	Immune response after tetanus/diphtheria booster
**study population**	Young (25-40 years.) and elderly (<60 years) healthy individuals, who are vaccinated against tetanus and diphtheria according to official recommendations
**study design**	The study is performed together with the Public Health Department, Federal State of Tyrol (Dr. Christoph Neuner). According to austrian vaccination recommendations adults (under 60 years of age) receive booster vaccinations against tetanus, diphtheria, pertussis and polio every 10 years there. For persons over 60 years of age the booster interval is shortened to 5 years for tetanus, diphtheria and pertussis. Persons scheduled for these booster vaccinations can participate in the study. In addition to the vaccination venous blood will be collected three times (total amount 95ml) and anamnestic data will be collected using questionnaires. The first blood collection (35ml) will be performed immediately before the vaccination by the vaccinating physician. .The second (after 7 days, 35ml) and third (after 28 days, 35ml) blood collection will be performed at the Institute for Biomedical Aging Research (IBA). In vitro analysis of the humoral and cellular immune response against tetanus and diphtheria is performed there (see 1.1). 100 healthy young (20-40 years) and 100 healthy elderly (>60 years) adults will be recruited. Serum will be isolated for quantification of tetanus- , diphtheria- and CMV-specific antibodies using ELISA-techniques. A certified diagnostic laboratory (Dr. Philadelphy) will analyze blood counts and several serum parameters (liver and kidney function, hormones, inflammatory parameters, metabolic parameters, markers of biological aging). In addition, peripheral blood mononuclear cells (PBMC) will be isolated and tetanus- and diphtheria-specific cellular immune responses (IFN-g, IL-5, IL-10 production by tetanus- or diphtheria-specific T cells) will be detected using ELISPOT-assays. In addition lymphocyte subpopulations will be quantified by flow cytometry.<br><br>.
**Aim of the study**	<u>Primary aim</u><br>To analyze age-related differences of the immune response against tetanus and diphtheria comparing young adults (booster interval 10 years) and older adults (booster interval 5 years).<br><br><u>Secondary aim</u><br>Identification of parameters predicting vaccination outcome
**Primary endpoint**	Quantification of vaccine-induced immune responses 4 weeks after vaccination.
**Participants**	Cohort 1: 100 individuals aged 25-40 years

	Cohort 2: 100 individuals aged >60 years
**Schedule**	<u>study related</u><br>planned start: May 2009<br>planned end: May 2010<br><br><u>participant related</u><br>treatment: single vaccination, last blood collection 28 days after vaccination
**Inclusion criteria**	Written informed consent<br><br>Cohort 1: Healthy individuals aged 25-40 years<br>Last vaccination against tetanus/diphtheria >10 years ago<br>Cohort 2: Healthy individuals aged >60 years<br>Last vaccination against tetanus/diphtheria >5 years ago
**Exclusion criteria**	• acute illness<br>• chronic inflammatory disease (Morbus Chron, Multiple Sclerosis etc.)<br>• chronic infection with HBV, HCV or HIV<br>• transplant recipient<br>• past chemotherapy<br>• immunosuppressive treatment<br>• pregnancy<br>• participation in another study within the last 4 weeks prior to inclusion
**Course of the study**	<u>Appointment 1 (day 0)</u><br>blood collection 1 (35ml), vaccination<br><u>Appoinment 2 (day 7)</u><br>blood collection 2 (35ml)<br><u>Appointment 3 (day 28)</u><br>blood collection 3 (25ml)
**Interventions and laboratory analyses**	*In vitro* analysis of the humoral and cellular immune response against tetanus and diphtheria.<br><br>Serum will be isolated for quantification of tetanus- , diphtheria- and CMV-specific antibodies using ELISA-techniques. A certified diagnostic laboratory (Dr. Philadelphy) will analyze blood counts and several serum parameters (liver and kidney function, hormones, inflammatory parameters, metabolic parameters, markers of biological aging). In addition, peripheral blood mononuclear cells (PBMC) will be isolated and tetanus- and diphtheria-specific cellular immune responses (IFN-g, IL-5, IL-10 production by tetanus- or diphtheria-specific T cells) will be detected using ELISPOT-assays. In addition lymphocyte subpopulations will be quantified by flow cytometry.
**Substance**	<u>Active component:</u> combined vaccine against tetanus, diphtheria, pertussis or against tetanus, diphtheria, pertussis, polio<br><u>Trade name:</u> Boostrix or. Boostrix Polio<br><u>Producer:</u> GlaxoSmithKlline AG

**Dosage**	<u>dosage: </u>1 dose of vaccine (0.5ml)<br><u>application:</u> intramuscular injection<br><u>duration:</u> single vaccination<br><br><u>Substance:</u><br>cohort 1 (young): Boostrix Polio<br>cohort 2 (old): Boostrix<br>according to official vaccination recommendations by the national authorities

1 Introduction

1.1 Background

With increasing age the immune system undergoes characteristic changes termed immunosenescence. These changes contribute substantially to the increased incidence and severity of infectious diseases observed in the elderly. Concomitantly, many vaccines, such as against influenza, hepatitis A and B, tetanus, diphtheria and tick-borne encephalitis (TBE) are less immunogenic and efficient in elderly persons. We have previously shown that tetanus- and TBE-specific antibody titers are lower in elderly compared to young adults, in whom the last vaccination dates back equally long. In addition, antibody concentrations decline faster in older persons. The success of booster vaccinations (tetanus, diphtheria, pertussis) depends on pre-existing antibody concentrations prior to the booster in elderly persons underlining the need of regular booster immunizations during adulthood.

Official recommendations (Oberster Sanitätsrats, www.bmgfj.gv.at) include regular vaccinations against tetanus, diphtheria, pertussis and polio every 10 years for adults. Since 2004 recommended booster intervals have been 5-years for adults older than 60 years for tetanus, diphtheria and pertussis. Intervals for polio booster vaccination are 10 years for all adult age groups, as antibody titers are usually sufficiently high and booster vaccination with inactivated polio vaccine is very successful due to efficient primary immunization with live-attenuated polio vaccine in the past. Participants of the study will be vaccinated according to these recommendations. Therefore young adults (25-40 years), whose last vaccination dates back more than 10 years will receive one dose of tetanus/diphtheria/pertussis/polio vaccine, whereas old adults (>60years) whose last vaccination dates back more than 5 years will receive one dose of tetanus/diphtheria/pertussis vaccine, but no polio component.

In this study we will analyze tetanus-and diphtheria-specific immune responses. Polio-specific immune responses are not determined as it has been demonstrated that adults, irrespective of age, respond very well to this vaccination. Analysis of pertussis-specific antibodies is technically difficult and it has been shown that antibodies are not suitable as correlates of protection. Therefore, pertussis-specific immune responses will also not be analyzed in this study.

1.2 Relevance of the study

No data are available analyzing tetanus- and diphtheria-specific immune responses of older adults receiving booster vaccination in 5-year intervals. It is unclear, whether older adults are adequately protected 5 years after their last vaccination and whether there are differences compared to young adults 10 years after their last vaccination. In addition, our study will show whether 5-year intervals for older adults result in immune responses similar to the ones achieved in young adults with vaccinations every 10 years.

Besides the specific immune response several biological parameters (liver and kidney function, hormones, inflammatory parameters, metabolic parameters, markers of biological aging) will be determined and correlations with vaccination success will be made in order to identify predictive markers for vaccination outcome. In addition the potential influence of latent CMV-infection on vaccine-induced immune responses will be analyzed. It has previously been demonstrated that concentrations of CMV-specific antibodies correlate with the outcome of influenza vaccination. No data are available addressing this issue for other vaccines, and a comparison of CMV-negative and CVM-positive individuals is missing.

1.3 Risk-Benefit-Assessment

All participants receive booster vaccinations according to official recommendations. No additional vaccination is administered. The vaccines used in this study (Boostrix and Boostrix Polio) are licensed in Austria. The participants will be informed about potential risks of the vaccine and of blood collections during the informed consent process. After vaccination local pain, redness and swelling might occur at the site of injection. Rarer events include fever, malaise, headache, dizziness or fatigue. At the site of venipuncture for blood collection skin irritation, bruising or minor pain might occur. The vaccines will be provided to the participants free of cost.

2 Aims of the study

2.1 Primary aim

This study analyzes vaccine induced tetanus- and diphtheria-specific humoral and cellular immune responses in adults of different age groups. Using the recommended vaccination schedule, questionnaires and three blood collections we aim to determine:

- whether older persons, whose last vaccination dates back 5 years are equally well protected against tetanus and diphtheria as young adults, whose last vaccination dates back 10 years.

- whether booster vaccination induces similar immune responses in old and young adults, if administered at intervals of 5 (old) or 10 (young) years, respectively.

2.2 Secondary aim

In addition we will address the question, whether biological parame ters (liver and kidney function, hormones, inflammatory parameters, metabolic parameters, markers of biological aging) correlate with vaccination outcome and might be suitable as predictive markers.

3 Study characteristics

3.1 Study design

uncontrolled, open Phase IV study

3.2 Primary endpoint

Quantification of vaccine-induced immune responses 28 days after vaccination

3.3 Participants

100 young (25-40 years, m/f 1:1) and 100 old (>60 years, m/f 1:1) healthy adults will be included in the study and will be vaccinated according to official recommendations. Therefore, young adults whose last vaccination dates back more than 10 years will receive one dose of tetanus/ diphtheria/

pertussis/ polio vaccine and old adults, whose last vaccination dates back more than 5 years will receive one dose of tetanus/ diphtheria/ pertussis vaccine (see 11).

3.4 Methods

Recruitment

The Public Health Department (Federal State of Tyrol) offers vaccination services including vaccination of older adults. Persons whose last vaccination against tetanus/ diphtheria/ pertussis/ polio dates back 5 years will receive a written reminder that they are due for a booster vaccination against tetanus/ diphtheria/ pertussis. In addition, they receive an invitation to participate in the study and to undergo 3 venous blood draws (total amount 95ml). Young adults will be recruited additionally using newspaper advertisements and announcements (e.g. at the University) asking for volunteers aged 25-40 years, whose last vaccination against tetanus and diphtheria dates back at least 10 years and who are therefore eligible for a booster vaccination against tetanus/ diphtheria/ pertussis/ polio. They will also undergo 3 blood draws (total amount 95ml). As compensation for their participation all volunteers will receive the vaccine free of cost.

Blood collection/vaccination/questionnaire

Three venous blood draws will be performed during the study. The first blood collection of 35ml will be performed immediately before vaccination. 5ml blood will be used for serum and 30ml heparinized blood will be used to isolate PBMC. The same procedure will be used for the second blood draw (7 days after vaccination). The last blood collection (28 days after vaccination) will include 15ml blood for serum and 10ml blood for lymphocyte counts and coagulation analysis. The total amount of blood is therefore 95ml.

The vaccines used are licensed combination vaccines. For the older cohort a vaccine against tetanus/ diphtheria/ pertussis (Boostrix, GlaxoSmithKline) and for the young cohort a vaccine against tetanus/ diphtheria/ pertussis/ polio (Boostrix Polio, GlaxoSmithKline) will be used according to official guidelines (see 1.1).

Using questionnaires we will collect information about underlying diseases, lifestyle (smoking, alcohol consumption, physical activity etc.)

Read-out parameters and laboratory methods

Analyses from serum:

Tetanus- and diphtheria-specific antibodies will be determined prior to and after vaccination using ELISA-methods. In addition, we will analyze biological parameters and investigate correlations with vaccination outcome. The aim of this analysis is the identification of predictive markers for vaccination outcome.

The following parameters will be determined:

- Liver and renal function

In order to exclude severe functional defects in liver and renal function, which might influence other parameters bilirubin, GGT, AST, ALT, electrolytes and creatinine will be quantified.

- Hormones

Several studies indicated a correlation of hormones and aging. Therefore concentrations fo testosterone, FSH, FT3, FT4, estradiol, progesterone and prolactin as well as PSA will be determined.

- inflammatory parameters

Subclinical inflammatory processes can be observed in older individuals and several inflammatory parameters have been described in the context of immunological function, frailty and 2-year mortality in the elderly. We therefore determine serum concentrations of IL-6, IL-10, C-reactive protein and neopterin as well as IDO-activity (calculated from tryptophan and kynurenine concentrations).

Fat metabolism

Active substances, which are produced by fat cells (adipokines) play a major role in metabolism. Low concentrations of leptin and adiponectin were described to be associated with increased mortality in the elderly. We will therefore determine serum concentrations of leptin and adiponectin.

- Latent CMV-infection

Several studies have shown that latent infection with Cytomegalovirus (CMV) aggravates age-related changes of the immune system. An impact of CMV on vaccination success has also been discussed in the literature We will therefore quantify CMV-specific antibodies in the serum using ELISA-methods.

- Parameters associated with cellular senescence

It has been demonstrated in several animal models that mutations of genes in the IGF (insuline-like growth factor) signaling pathway influence lifespan. This pathway is believed to play a major role in cellular senescence. We will determine serum concentrations of IGF, IGFBP-3 (IGF-binding protein) und IGFBP-6. In addition dkk-3 (Dickkopf-3) and TL1A (TNF superfamily ligand 1A) concentrations will be analyzed as these proteins are investigated in the context of aging epithelial and endothelial cells at our institute.

Analyses from whole blood

Lymphocyte counts and coagulation analysis will be performed from whole blood by a certified diagnostic laboratory.

Analyses from isolated PBMC

The cellular immune response against tetanus and diphtheria will be analyzed using ELISPOT-methodology. CD4+ T cells are stimulated with tetanus and diphtheria toxoid and their production of cytokines (IFN-g, IL-5, IL-10) will be detected on the single-cell level. In addition we will analyze individual antibody-secreting B cells.

Surface markers of lmyphocytes (CD3, CD4, CD5, CD8, CD14, CD16, CD19, CD20, CD22, CD25, CD27, CD28, CD38, CD45RA, CD62L, CD138 and FoxP3) will be determined by flow cytometry in order to identify different cell types (T cells, B cells, monocytes, NK cells) and T and B cell subpopulations (naïve, memory, effector T cells, plasma cells, plasmablasts). We will also determine telomere length of PBMC using Flow-FISH. Telomere length is used to analyze prior cell proliferation as telomeres shorten with every cell divisioni. It has been shown that highly differentiated, senescent T cells have short telomers and that telomere length of PBMC is associated with increased mortality.

3.5 Schedule

The study is scheduled for 1 year and will start in May 2009.

4 Study population

4.1 Inclusion criteria

Healthy persons giving written informed consent can participate.

Cohort 1: individuals aged 25-40 years; last vaccination against tetanus/diphtheria >10 years ago
Cohort 2: individuals aged >60 years; last vaccination against tetanus/diphtheria >5 years ago

4.2 Ausschlusskriterien

Persons suffering from acute illness, chronic inflammatory disease (Morbus Chron, Multiple Sclerosis etc.) or chronic infection with HBV, HCV or HIV are excluded. In addition transplant recipients, persons who had chemotherapy in the past or are currently under immunosuppressive treatment will not be included. According to legal requirements (AMG) pregnant woman will not be included in the study. Pregnancy testing will be performed prior to inclusion into the study (only for females of cohort 1) in order to exclude pregnancy.

5 Substance

5.1 Description of study substance

Boostrix:

Boostrix (GlaxoSmithKline AG) is licensed for booster vaccination against tetanus, diphtheria and pertussis in persons above 4 years of age. The vaccine will be purchased in single doses at a local pharmacy (Apotheke zum Tiroler Adler, Innsbruck). A single dose contains 0.5ml suspension in a ready-to-use syringe. The antigens (tetanus toxoid, diphtheria toxoid, pertussis antigens) are adsorbed to aluminum hydroxide and aluminum phosphate. In addition the vaccine contains formaldehyde, 2-phenoxyethanol, polysorbat 80, sodium chloride, glycine and water for injection. The vaccines are stored at 2-8°C. The date of expiry is given on the individual packages.

Boostrix Polio:

Boostrix Polio (GlaxoSmithKline AG) is licensed for booster vaccination against tetanus, diphtheria, pertussis and polio in persons above 4 years of age. The vaccine will be purchased in single doses at a local pharmacy (Apotheke zum Tiroler Adler, Innsbruck). A single dose contains 0.5ml suspension in a ready-to-use syringe. The antigens (tetanus toxoid, diphtheria toxoid, pertussis antigens and inactivated polio virus) are adsorbed to aluminum hydroxide and aluminum phosphate. In addition the vaccine contains medium 199, neomycin, polymyxin, polysorbat 80, sodium chloride, glycine and water for injection. The vaccines are stored at 2-8°C. The date of expiry is given on the individual packages.

Both vaccine may not be administered to persons with known hypersensitivity reactions after prior doses of these vaccines or to one of the vaccine components.

5.2 Adverse events

The patient informations contain a detailed list of adverse events. The following shows frequent and very frequent adverse events. Rarer events are listed in the patient information.

Boostrix:

In adults pain, redness and swelling can occur at the site of injection. In addition, headache, malaise, vomiting, fatigue, drowsiness or fever might occur.

Boostrix Polio:

In adults pain, redness and swelling can occur at the site of injection. In addition, fatigue, fever, malaise, headache, abdominal pain, nausea or vomiting might occur.

5.3 Treatment plan

5.3.1 Application

single dose of vaccine

5.3.2 Emergency response

Vaccination will be performed by experienced physicians and equipment necessary for emergency treatment will be available.

5.3.3 Other medication

Participants are allowed to continue their regular medication.

5.4 Drug Accountability

Vaccines will be purchased at a local pharmacy, will be stored at the Institute for Biomedical Aging Research and will be supplied to the Department of Public Health on demand. The number of doses purchased and used will be documented. Storage conditions will be 2-8°C and a temperature log as well as a drug accountability log will be available in the study documents.

6 Course of the study

6.1 Appointment 1 (day 0)

All participants will be informed about the course and the aims of the study. This information will be given personally by the physician performing the vaccination and in written form by the “informed consent form”. After discussion of all open questions the participant signs and dates two copies of the “informed consent form”. One copy stays with the investigator, the other copy is given to the participant. Demographic data will be collected and inclusion and exclusion criteria will be checked. After that blood collection (35ml) and vaccination will be performed.

6.2 Appointment 2 (day 7)

During the second visit information about underlying diseases and life-style (physical activity, smoking, alcohol consumption) will be collected using a questionnaire. The second blood draw (35ml) will be performed. In addition local reactions to the first blood draw as well as local and systemic adverse events of the vaccination will be inquired and documented.

6.3 Appointment 3 (day 28)

During the third visit 25ml blood will be collected. In addition local reactions to the second blood draw as well as local and systemic adverse events of the vaccination that might have occurred after the second visit will be inquired and documented.

6.3.1 Laboratory analysis

Blood samples collected during the first appointment (day 0) at the Public Health Department are transported each day to the Institute for Biomedical Aging Research, where laboratory analyses of antibody concentrations (ELISA), cellular immune responses (ELISPOT) and lymphocyte populations (flow cytometry) are performed. The second blood collection (day 7) will take place at the Institute for Biomedical Aging Research. Therefore transportation is not necessary. Antibody concentrations, cellular immune responses and lymphocyte populations will again be determined. As this study investigates booster immunization these analyses are performed prior to and after vaccination. Participants already have pre-existing immune responses against tetanus and diphtheria, which are enhanced by the vaccination. This booster effect will be studied. In preliminary experiments it has been shown that specific T cells and antibody-secreting B cells are detectable in the periphery 7 days after vaccination. After that their numbers in the peripheral blood decrease. Therefore, the best time point for the detection of cellular immune responses is at day 7 after vaccination.

The third blood collection (day 28) will also be performed at the Institute for Biomedical Aging Research. Several health parameters (lymphocyte count, coagulation, bilirubin, GGT, AST, ALT, electrolytes, creatinine, testosterone, FSH, FT3, FT4, estradiol, progesterone, prolactin, CRP, IGF) will be measured by a certified diagnostic laboratory (Dr. Philadelphy). Samples are collected by the laboratory every day. At the Institute for Biomedical Aging Research PSA, IGFBP-3, IGFBP-6, leptin, adiponectin, dkk-3, TLA1A, IL-6 and IL-10 will be quantified. In addition IDO activity (calculated from tryptophan and kynurenine concentrations) and CMV-specific antibodies will be determined. The increase in tetanus- and diphtheria- specific antibodies after vaccination takes more time than the cellular immune response. Maximum antibody concentrations are reached 28 days after vaccination. Therefore antibody concentrations will again be determined at that time-point facilitating comparison of our data with previously published results. Almost all studies investigating antibody responses use this time-point for their analysis.

6.4 Drop-out

Participants can drop-out from the study by their own request at any given time and without explanation of their reasons. In addition the investigator can initiate the drop-out of participants in case of medical concerns. Drop-out from the study will not negatively impact future medical treatment of the participants.

6.5 Premature termination

No criteria are defined as premature termination of the study is not anticipated.

7 Documentation of adverse events

During the 2nd and 3rd visit local and systemic adverse events after vaccination as well as discomfort or other reactions at the site of venipuntcture will be inquired and documented in the case report form (CRF). Severe and unexpected adverse events will be reported to the ethics committee and the local authorities according to legal requirements.

8 Documentation

The principal investigator has the responsibility to ensure execution of the trial in accordance to GCP guidelines, legal requirements (Arzneimittelgesetz, AMG) and the study protocol and to guarantee correct data documentation in the CRF. All data obtained during the study must be documented in the CRF by authorized personnel. This applies also to participants, who are excluded from the study.

A “patient identification list” containing participant code, full name, date of birth and date of inclusion in the study will be generated by the principal investigator in order to enable identification of participants at later time points.

8.1 Case report form (CRF)

All data and results will be documented in CRFs designed for the study.

CRFs will be filled in with a black pen and corrections will be made in a way that the original text is still legible (no white-out allowed). Corrections need to be made by authorized personnel and must be dated and signed. Missing data or results need to be clearly marked (n.a. or n.d.) and explantations should be documented

The principal investigator will ensure that all data are immediately, completely and correctly documented in a legible way in the CRF in accordance with health records. Plausibility and completeness of the original CRFs will be reviewed by the monitor.

8.2 Principal investigator´s folder

All documents required for the study will be collected and stored in the PI folder. Completeness and timeliness of the PI folder will be reviewed by the monitor. The PI folder has to be stored for 15 years after the end or termination of the study.

8.3 Data storage

8.3.1 Sponsor´s responsibilities

All relevant documents of the study must be stored by the sponsor for 10 years after the end or termination of the study. It is the sponsor´s responsibility to archive the relevant documents according to legal requirements.

8.3.2 Prinicipal investigator´s responsibilities

Documents related to the study or to the study medication (questionnaires, informed consent forms, drug accountability documents and other relevant material) must be stored by the principal investigator for 10 years. Medical records and other primary data must be stored for as long as possible in accordance with the institution´s or hospital´s practice.

9 Monitoring

9.1 Monitoring

The principal investigator agrees that the monitor reviews all data to ensure adequate documentation and compliance with the study protocol. In addition, the principal investigator agrees to cooperate with the monitor and to enable access to all relevant information whenever necessary. This includes access to all documents related to the study. The monitor will also be eligible to review data in regular, fixed intervals and to validate them in accordance with SOPs and ICH-GCP guidelines in order to ensure compliance with the study protocol and timely documentation. For this purpose all medical records used for documentation within the CRF and the data bank will be reviewed. Participants agree to this review by signing the informed consent form.

Further responsibilities of the monitor are:

-

Weitere Aufgaben des Monitors sind:

- to ensure suitability of the infrastructure (instruments, participants, storage of study documents and materials…)
- to review completeness and timeliness of the principal investigator´s folder
- to validate original data
- to ensure adequate documentation and reporting of SAEs
- to ensure adequate and safe storage of study medications and to check their date of expiry
- drug accountability (count of unused medication)

All data is strictly confidential and the monitor agrees to respect the general right of the participants for privacy and data protection.

10 Data management

The CRF contains information on acute and chronic diseases, as well as on allergies, vaccination history and current medication. In addition, dates of informed consent and blood collections are denoted and results of laboratory analyses are documented. The CRF does not contain any personal information (name,date of birth etc.), but only the anonymized participant code. Identification of this code is only possible using the “personal data form”, which will be archived separately at the Institute for Biomedical Aging Research and will be strictly confidential. Anonymized participant codes will be used for data analysis, documentation and publication.

CRFs and signed informed consent forms will be archived at the Institute for Biomedical Aging Research. Data will be stored and analyzed by the Institute for Biomedical Aging Research using Excel and SPSS software.

11 Statistics

11.1 Sample size estimation

This explorative study aims to identify predictive markers of vaccination outcome for tetanus and diphtheria. Potential predictors are the age group (20-40 years vs. 60-100 years) and several biological parameters.

The sample size estimation is based on an explorative multiple logistic regression with vaccination success being the dependent variable and age and biological parameters as independent variables.

Given an estimation of 10 effective predictors (age, gender, 8 biological parameters), a vaccination success rate of 50% and a ratio of 1:1 for young and old participants the required sample size is calculated to be 200 (100 participants in each age group) based on the general regression model of Harrell (approx. 10 events required per predictor identified).

11.2 Statistical methods

Dependent variable

The primary dependent variable is the Die primäre Zielgröße der Analysen ist die Impferfolgswahrscheinlichkeit.

Data analysis

Explorative data analysis will be performed using multiple logistic regression. Variables will be selected in two steps. Firstly, potential predictors will be selected using univariate logistic regression ($p<0.20$). Based on these results the final prediction model will be calculated using multiple logistic regression and forward selection methods. Odd´s ratios and 95% confidence intervals will be estimated. Interactions between age and biological parameters and between biological parameters will be explored. A predictive score for vaccination success will be calculated and reported as c-value.

12 Reporting

12.1 Study report

All information regarding the study is strictly confidential. The statistical analysis and the final study report will be provided by the Institute for Biomedical Aging Research and the Institute for Public Health, Medical Decision Making and HTA and will be reviewed and signed by all other persons involved.

12.2 Publication

The results of this study will be published only after consent of all cooperation partners. Anonymity and data safety must be ensured for all information related to study participants.

13 Ethical, legal and administrative aspects

13.1 Responsibilities of the sponsor and the principal investigator

According to legal requirements (AMG) the sponsor is responsible for the initiation, organization and funding of the study. Sponsor and principal investigator ensure that the study is conducted in accordance with current laws and regulations, ICH-GCP guidelines (1996), the declaration of Helsinki (1996) as well as with the regulations of the AMP and the GCP act (2004). The principal investigator agrees to the signed study protocol.

It is the responsibility of the principal investigator:

- to understand the properties of the investigated drug according to the Summary of Product Characteristics
- to understand and to comply with the study protocol
- to ensure sufficient time and capacities for the conduct of the study
- to ensure correct collection and documentation of data and reporting
- to guarantee access to all data for review by the sponsor, the monitor or responsible authorities for audit and inspection
- to ensure that all information regarding participants and all information provided by the sponsor are strictly confidential and that all persons involved in the trial comply with this requirement.

13.2 Ethical permission and registration at the competent authorities

Ethical permission is requested by the ethics committee of the Innsbruck Medical University and the trial is registered at the competent authorities (Bundesamt für Sicherheit im Gesundheitswesen) by Prof. Beatrix Grubeck-Loebenstein on behalf of the sponsor.

13.3 Informed consent

Prior to enrollment each participant has to give written informed consent. The nature, meaning and consequences of the study have to be fully explained by the investigator orally and in writing in a way, which is understandable for the participant. The content of this explanation is documented in the informed consent form. The consent of the participant requires his dated signature and the dated signature of the investigator. The participant receives one copy of the consent form, a second signed copy is archived in the principal investigator´s file.

It is explicitly pointed out, that no study-related examinations may be carried out prior to effectual written informed consent.

13.4 Insurance

The following insurance has been procured by the sponor as legally required by the AMG for all study participants:

Name of the company:	HDI Versicherung
Policy number:	1375586
Address :	Edelsinnstr. 7-11, 1120 Wien
Telefon:	0509-05 501 420
Telefax:	0509-05 501 420

Within this policy study-related damages to health are covered up to a maximum of € 500,000 per participant. This policy covers all eventual damages, which are inflicted directly or indirectly by the study substance or study-related procedures.

Participants are eligible to get access to the terms and conditions of the policy and to obtain a copy.

The participants are made aware that the policy does not cover accidents that might occur on the way to or from the study center.

13.5 Data protection and confidentiality

Collection, transfer, storage and analysis of study-related personal data are performed in compliance with legal requirements (Sächsisches Datenschutzgesetz, Bundesdatenschutzgesetz). Prerequisite is the voluntary written informed consent as given on the informed consent form prior to enrollment. The participants are informed about the following during the consent procedure:

1. Data collected during this study are documented in paper forms and/or electronically, are strictly confidential and are only transferred in anonymized form to the following parties:

   – the sponsor of the study for scientific analysis and evaluation of adverse events
   – the competent national authorities, the ethics committee of the Medical University Innsbruck, the European regulatory data bank for review of correct conduct of the study and for evaluation of adverse events.

2. If necessary for evaluation of the study personal data can be reviewed by authorized persons (monitor, auditor) assigned by the sponsor and sworn to secrecy. For this process the principal investigator is relieved of his medical confidentiality.

3. The consent for collection and analysis of personal data within this study is irrevocable. The participant is informed that he can terminate his participation in the study at any time without stating the reason and without negative consequences for him. In case of retraction of consent data collected until this time point will be further utilized in anonymized form, if this is necessary to assess the effect of the study substance and under the condition that interests of the participant meriting protection are not compromised.
