# Supplementary material for: Recall Responses to Tetanus and Diphtheria Vaccination Are Frequently Insufficient in Elderly Persons
Source: PLoS One. 2013 Dec 11;8(12):e82967. doi: 10.1371/journal.pone.0082967 (PMC3859625; doi:10.1371/journal.pone.0082967)
Supplement: Protocol S2 — Trial protocol (german). (PDF) [file pone.0082967.s003.pdf]

PRÜFPLAN/GESAMTPROTOKOLL

Version 2, 23.03.2009

Vertraulich

Titel der Studie	Humorale und zelluläre Immunantwort gegen Tetanus und Diphtherie nach Auffrischungsimpfungen
Kurzbezeichnung	Immunantwort nach Tetanus/Diphtherie-Booster
Sponsor	Institut für Biomedizinische Alternsforschung<br>Rennweg 10<br>6020 Innsbruck
Prüfplancode des Sponsors	TetDip-2009
EudraCT-Nummer	2009-011742-26

SPONSOR DER STUDIE

Institut für Biomedizinische Alternsforschung
Rennweg 10
6020 Innsbruck

Verschwiegenheitspflicht

Die in diesem Protokoll zur Verfügung gestellten Informationen sind streng vertraulich und werden den Prüfärzten, potentiellen Prüfärzten, Gesundheitsbehörden und Ethikkommissionen zur Einsicht beziehungsweise zur Überprüfung zur Verfügung gestellt. Eine Veröffentlichung ohne vorherige schriftliche Zustimmung ist nicht gestattet. Eine Ausnahme hiefür stellt die Einholung einer Einverständniserklärung von einem potentiellen Studienteilnehmer dar. Nach Unterschrift sind die Regelungen dieses Protokolls für alle Parteien bindend.

Erklärung des Sponsors

Das vorliegende Studienprotokoll wurde einer kritischen Überprüfung unterzogen. Der Inhalt ist in Übereinstimmung mit der derzeitigen Nutzen-Risiko-Einschätzung für die beschriebenen Methoden sowie mit den moralischen, ethischen und wissenschaftlichen Prinzipien der Guten Klinischen Praxis, der letztgültigen Version der Deklaration von Helsinki und den lokalen Gesetzen und Verordnungen.

**Prof. Beatrix Grubeck-Loebenstein**<br>*Vertreterin des Sponsors (Institut für Biomedizinische Alternsforschung)*<br>*und*<br>*Principal Investigator der Studie*	Innsbruck, 26.03.09<br>Ort, Datum, Unterschrift

Die oben Unterzeichnenden bestätigen, dass Sie das vorliegende Studienprotokoll gelesen haben und bestätigen, dass das Studienprotokoll alle notwendigen Informationen enthält, die für die Studiendurchführung notwendig sind. Sie bestätigen darüber hinaus, die Studie gemäß dem vorliegenden Studienprotokoll durchzuführen. Es wird vereinbart, dass jede bis dato nicht veröffentlichte Information der strengsten Geheimhaltung obliegt.

UNTERSCHRIFTEN

Prüfpräparat:	**Boostrix und Boostrix Polio**
Studientitel:	Humorale und zelluläre Immunantwort gegen Tetanus und Diphtherie nach Auffrischungsimpfungen
Kurztitel :	Immunantwort nach Tetanus/Diphtherie-Booster
EUDRACT Number:	2009-011742-26

Erklärung des Biometrikers, des Studienkoordinators und des verantwortlichen Monitors:

Ich habe dieses Studienprotokoll gelesen und stimme dem Inhalt dieses Studienprotokolls zu. Ich bestätige, dass es alle zur Studiendurchführung notwendigen Informationen enthält. Ich bin damit einverstanden, die Studie gemäß den Ausführungen dieses Protokolls durchzuführen. Insbesondere werde ich die moralischen, ethischen und wissenschaftlichen Prinzipien der Guten Klinischen Praxis (Good Clinical Practice – GCP), die aktuelle Version der Deklaration von Helsinki, lokale Gesetze und Bestimmungen sowie die maßgeblichen regulatorischen Anforderungen befolgen.

Ich werde Veränderungen, die die Verantwortlichkeit jedes Einzelnen der Unterzeichnenden betreffen, unverzüglich anzeigen.

Ich bestätige, jede Information und alle Unterlagen, die vorab noch nicht publiziert wurden, strengstens vertraulich zu behandeln. Diese Unterlagen beinhalten das Studienprotokoll, Prüfbögen und andere wissenschaftliche Daten.

**Prof. Uwe Siebert**<br>*Biometriker*	Hall, 26.03.09<br>Ort, Datum, Unterschrift
**Dr. Birgit Weinberger**<br>*Studienkoordinator*	Innsbruck, 26.03.09<br>Ort, Datum, Unterschrift
**KKS Innsbruck**<br>*Verantwortlicher Monitor*	Innsbruck, 26.03.09<br>Ort, Datum, Unterschrift

Beteiligte Personen/Institutionen

Sponsor:

Institut für Biomedizinische Alternsforschung, vertreten durch
Prof. Beatrix Grubeck-Loebenstein
Rennweg 10
6020 Innsbruck
Tel: 0512/583919-10
Fax: 0512/583919-8
beatrix.grubeck@oeaw.ac.at

Labor:

Dr. Birgit Weinberger
Rennweg 10
6020 Innsbruck
Tel: 0512/583919-13
Fax: 0512/583919-8
birgit.weinberger@oeaw.ac.at

Labor Dr. Philadelphy
Andreas-Hofer-Str. 28
6020 Innsbruck
Tel:0512/58021
Fax: 0512/58021-20
labor.philadelphy@phillab.at

Impfung:

Landessanitätsdirektion für Tirol
Dr. Christoph Neuner
Eduard-Wallnöfer-Platz 3
6020 Innsbruck
Tel: 0512/508-2662
Fax: 0512/508-2665
sanitaetsdirektion@tirol.gv.at

Studienkoordination:

Dr. Birgit Weinberger

Datenmanagement:

Dr. Birgit Weinberger

Monitoring:

Koordinierungszentrum für Klinische Studien (KKS)
Anichstr. 35
6020 Innsbruck
Tel: 0512/900370086
Fax: 0512/900370086
ctc@i-med.ca.at

Biometrie:

Prof. Uwe Siebert
UMIT
Department of Public Health, Medical Decision Making and Health Technology Assessment
Eduard-Wallnöfer-Zentrum 1
6060 Hall
Tel: 050/8648-3939
Fax: 050/8648-673930
public-health@umit.at

Synopsis

**Sponsor**	Institut für Biomedizinische Alternsforschung
**Titel**	Humorale und zelluläre Immunantwort gegen Tetanus und Diphtherie nach Auffrischungsimpfung
**Kurzbezeichnung**	Immunantwort nach Tetanus/Diphtherie-Booster
**Zielpopulation**<br>(oder Indikation)	Junge (25-40J.) und ältere (<60J.) gesunde Personen, die gemäß den offiziellen Impfempfehlungen gegen Tetanus und Diphtherie geimpft werden
**Studiendesign**	Diese Studie wird in Zusammenarbeit mit der Landessanitätsdirektion (Leitung: Dr. Christoph Neuner) durchgeführt. Gemäß den Empfehlungen des Obersten Sanitätsrats erhalten dort Erwachsene (unter 60 Jahre) alle 10 Jahre Auffrischungsimpfungen gegen Tetanus, Diphtherie, Pertussis und Polio. Für Personen über 60 Jahren sind die Impfabstände für Tetanus, Diphtherie und Pertussis auf 5 Jahre verkürzt. Personen, die -unabhängig von dieser Studie- Impfungen gegen Tetanus/Diphtherie/Pertussis/Polio (junge Probanden) bzw. Tetanus/Diphtherie/Pertussis (alte Probanden) erhalten, können an der Studie teilnehmen. Es erfolgen dann drei Blutentnahmen (insgesamt 95ml) und eine Anamnese mittels eines Fragebogens. Die erste Blutentnahme (35ml) wird unmittelbar vor der Impfung durch den impfenden Arzt der Landessanitätsdirektion durchgeführt. Die zweite (7 Tage nach Impfung, 35ml) und dritte (28 Tage nach Impfung, 25ml) Blutentnahme finden am Institut für Biomedizinische Alternsforschung (IBA) ÖAW (Prof. Grubeck-Loebenstein) statt. Dort erfolgt auch die *in vitro* Untersuchung der humoralen und zellulären Immunantwort gegen Tetanus und Diphtherie. Immunantworten gegen Polio und Pertussis werden nicht untersucht (siehe 1.1) Dafür sollen 100 gesunde, junge Probanden (20-40 Jahre) und 100 gesunde ältere Probanden (≥60 Jahre) in die Studie eingeschlossen werden.<br><br>Aus dem Blut wird Serum zur Quantifizierung der Antikörpertiter (gegen Tetanus, Diphtherie und CMV) mittels ELISA gewonnen. In einem zertifizierten Routine-Diagnostiklabor (Labor Dr. Philadelphy) wird ein Blutbild erstellt und verschiedene Parameter aus Serum analysiert (Leber- und Nierenfunktion, Hormone, Entzündungsparameter, Parameter des Fettstoffwechsels, Parameter, die mit der Zellalterung assoziiert sind). Außerdem werden periphere mononukleäre Lymphozyten (PBL) isoliert und die zelluläre Immunantwort gegen die Impfungen mittels ELISPOT-Assays analysiert. Dabei wird die Produktion der Zytokine Interferon-gamma, Interleukin 5 und Interleukin 10 durch T-Zellen, die mit Tetanus- und Diphtherietoxoid stimuliert wurden auf Einzelzellniveau detektiert. Zusätzlich werden von allen Probanden verschiedene Lymphozytenpopulationen mittels FACS-Analyse quantifiziert.
**Studienziele**	<u>Primäres Studienziel</u><br>Analyse, ob die Immunantwort gegen Tetanus und Diphtherie bei älteren Personen, die 5 Jahre nach der letzten Impfung eine Auffrischung

	erhalten, Unterschiede aufweist zu der entsprechenden Immunantwort bei jungen Erwachsenen, die alle 10 Jahre eine Auffrischung erhalten.<br><br>Sekundäres Studienziel<br>Identifizierung von biologischen Parametern, die als prädiktive Marker für den Impferfolg dienen können.
**Primärer Endpunkt**	Quantifizierung der impfinduzierten Immunantwort 4 Wochen nach der Impfung
**Probandenzahl**	Gruppe 1: 100 Personen im Alter von 25-40 Jahren<br><br>Gruppe 2: 100 Personen über 60 Jahre
**Zeitplan**	Studienbezogen<br>Geplanter Beginn : Mai 2009<br>Geplantes Studienende : Mai 2010<br><br>Probandenbezogen<br>Behandlungsdauer: einmalige Impfung, letze Blutentnahme 28 Tage nach der Impfung
**Einschluss-kriterien**	Schriftliche Einwilligung des Probanden nach erfolgter Aufklärung<br><br>Gruppe 1: Gesunde Personen im Alter von 25-40 Jahren<br>Letzte Tetanus/Diphtherie-Impfung vor 10 Jahren<br><br>Gruppe 2: Gesunde Personen über 60 Jahre<br>Letzte Tetanus/Diphtherie-Impfung vor 5 Jahren
**Ausschluss-kriterien**	• Akute Erkrankung<br>• Chronische entzündliche Krankheiten (Morbus Chron, Multiple Sklerose etc.)<br>• Chronische Infektion mit HBV, HCV oder HIV<br>• Transplantatempfänger<br>• Personen, die sich in der Vergangenheit einer Chemotherapie unterzogen haben<br>• Personen, die mit immunsuppressiven Medikamenten behandelt werden<br>• Schwangere Personen<br>• Teilnahme des Probanden an einer anderen klinischen Studie innerhalb der letzten 4 Wochen vor dem Einschluss
**Ablauf der Studie**	Termin 1 (Tag 0)<br>Blutentnahme 1 (35ml), Impfung<br>Termin 2 (Tag 7)<br>Blutentnahme 2 (35ml)<br>Termin 3 (Tag 28)<br>Blutentnahme 3 (25ml)
**Studienbezogene Verfahren und**	*In vitro* Untersuchung der humoralen und zellulären Immunantwort gegen Tetanus und Diphtherie. Aus dem Blut wird Serum zur Quantifizierung der Antikörpertiter (gegen Tetanus, Diphtherie und CMV)

**Laboruntersuchungen**	mittels ELISA gewonnen. In einem zertifizierten Routine-Diagnostiklabor wird ein Blutbild erstellt und verschiedene Parameter aus Serum analysiert (Leber- und Nierenfunktion, Hormone, Entzündungsparameter, Parameter des Fettstoffwechsels, Parameter, die mit der Zellalterung assoziiert sind). Außerdem werden periphere mononukleäre Lymphozyten (PBL) isoliert und die zelluläre Immunantwort gegen die Impfungen mittels ELISPOT-Assays analysiert. Dabei wird die Produktion der Zytokine Interferon-gamma, Interleukin 5 und Interleukin 10 durch T-Zellen, die mit Tetanus- und Diphtherietoxoid stimuliert wurden auf Einzelzellniveau detektiert. Zusätzlich werden von allen Probanden verschiedene Lymphozytenpopulationen mittels FACS-Analyse quantifiziert.
**Prüfmedikation**	Wirkstoff: Kombinationsimpfstoff gegen Tetanus, Diphtherie und Pertussis bzw. gegen Tetanus, Diphtherie, Pertussis und Polio<br>Handelsname: Boostrix bzw. Boostrix Polio<br>Hersteller: GlaxoSmithKlline AG
**Behandlungsplan**	Dosis: 1 Dosis Impfstoff (0,5ml)<br>Applikationsform: intramuskuläre Injektion<br>Dauer der Therapie: einmalige Impfung<br><br>Prüfmedikation:<br>Gruppe 1 (jung) erhält Boostrix Polio<br>Gruppe 2 (alt) erhält Boostrix<br>gemäß den offiziellen Impfempfehlungen des Obersten Sanitätsrats

1 Einleitung

1.1 Hintergrund der Studie

Im Alter kommt es zu charakteristischen Veränderungen des Immunsystems, die als Immunseneszenz bezeichnet werden. Sie sind einer der Hauptgründe dafür, daß im Alter die Inzidenz und Schwere von Infektionskrankheiten zunimmt. Gleichzeitig zeigen viele Impfungen, z.B. Influenza, Hepatitis A und B, Tetanus, Diphtherie und FSME (Frühsommer-Meningoencephalitis) bei älteren Menschen eine verringerte Wirksamkeit. Arbeiten in unserer Gruppe zeigen, daß ältere Menschen niedrigere Antikörper-Titer gegen Tetanus und FSME haben als junge Erwachsene, bei denen die letzte Impfung gleich lang zurückliegt. Außerdem sinken die Antikörper-Titer bei älteren Personen in den Jahren nach der Impfung schneller ab. Der Erfolg einer Auffrischungsimpfung (Tetanus, Diphtherie und Pertussis) hängt bei älteren Personen von den Antikörper-Titern vor der Auffrischung ab, was die Notwendigkeit von regelmäßigen Auffrischungsimpfungen im Erwachsenenalter unterstreicht.

Für Erwachsene wird eine regelmäßige Impfung gegen Tetanus, Diphtherie, Pertussis und Polio alle 10 Jahre empfohlen (Empfehlung des Obersten Sanitätsrats, www.bmgfj.gv.at). Seit 2004 wird für Personen über 60 Jahren eine Verkürzung der Impfabstände für Tetanus, Diphtherie und Pertussis auf 5 Jahre empfohlen. Die Abstände für Polio-Auffrischung sind auch für ältere Personen bei 10 Jahren belassen worden, da Personen aller Altersgruppen ausreichende Antikörper-Titer besitzen und die Immunantwort gegen Polio sehr gut auf Auffrischungsimpfungen anspricht. Dies ist darauf zurückzuführen, dass in der Vergangenheit mit einem attenuierten Lebendimpfstoff, der eine sehr effektive Immunantwort induziert, gegen Polio geimpft wurde. Für diese Studie werden Probanden herangezogen, die -gemäß der Impfempfehlung-Auffrischungsimpfungen gegen Tetanus, Diphtherie, Pertussis und Polio erhalten. Aufgrund der unterschiedlichen Impfempfehlungen für junge und ältere Erwachsene werden bei dieser Studie junge Erwachsene (25-40 Jahre) teilnehmen, bei denen die letzte dieser Auffrischungsimpfungen 10 Jahre zurückliegt und die entsprechend eine Impfung gegen Tetanus, Diphtherie, Pertussis und Polio erhalten. Die Gruppe der älteren Probanden (>60 Jahre) umfasst Personen bei denen die letzte dieser Auffrischungsimpfungen 5 Jahre zurückliegt und die deshalb nur gegen Tetanus, Diphtherie und Pertussis, nicht jedoch gegen Polio geimpft werden.

Im Rahmen dieser Studie wird die Immunantwort gegen Tetanus und Diphtherie nach der Auffrischungsimpfung untersucht. Die Polio-spezifische Immunantwort wird nicht analysiert, da bereits gezeigt wurde, dass Personen jeden Alters sehr gut auf diese Auffrischung reagieren. Die Analyse von Pertussis-spezifischen Antikörpern ist technisch nur sehr schwierig möglich. Außerdem wurde gezeigt, dass Antikörper-Titer als Korrelat für Schutz vor der Erkrankung nicht geeignet sind. Deshalb ist die Immunantwort gegen Pertussis ebenfalls nicht Gegenstand dieser Studie.

1.2 Notwendigkeit der Durchführung der Studie

Bisher gibt es keine Daten zur Tetanus- und Diphtherie-spezifischen Immunantwort von älteren Personen, die 5 Jahre nach der letzten Auffrischungsimpfung wieder geimpft werden. Es ist unklar, ob ältere Personen 5 Jahre nach der Impfung noch ausreichend geschützt sind und ob es in Bezug auf die noch vorhandene Immunantwort Unterschiede zu jungen Erwachsenen, die vor 10 Jahren das letzte Mal geimpft wurden, gibt. Außerdem wird unsere Studie zeigen, ob ältere Personen, die alle 5 Jahre geimpft werden auf diese Auffrischung genauso gut reagieren, wie junge Personen bei denen der Impfabstand 10 Jahre beträgt.

Neben der Immunantwort werden bei den Probanden eine Reihe von biologischen Parametern (Leber- und Nierenfunktion, Hormone, Entzündungsparameter, Parameter des Fettstoffwechsels, Parameter, die mit der Zellalterung assoziiert sind) untersucht. Ziel ist es Parameter zu identifizieren, die mit dem Impferfolg korrelieren und als prädiktive Marker für den Impferfolg geeignet sind. Auch ein möglicher Einfluss einer latenten CMV-Infektion auf die Immunantwort nach Impfungen soll untersucht werden. Es wurde gezeigt, daß die Konzentration von CMV-Antikörpern mit dem Erfolg von Influenza-Impfungen korreliert. Daten zu anderen Impfungen und eine Gegenüberstellung von CMV-seronegativen und CVM-seropositiven Probanden liegen noch nicht vor.

1.3 Nutzen-Risiko-Abschätzung

Alle Probanden nehmen im Rahmen ihrer Routine-Auffrischungsimpfungen (gemäß den Empfehlungen des Obersten Sanitätsrats) an dieser Studie teil. Es werden also keine Impfungen ausschließlich für diese Studie verabreicht. Die verwendeten Impfstoffe (Boostrix und Boostrix Polio) sind in Österreich zugelassen. Über die möglichen Risiken der Impfung und der Blutabnahme wird der Spender im Rahmen seiner Einverständniserklärung informiert Nach der Impfung kann es zu lokalen Schmerzen, Rötungen und Schwellungen an der Injektionsstelle kommen. Seltener kommt es zu Fieber, Unwohlsein, Kopfschmerzen, Schwindel oder Müdigkeit. Nach der Blutentnahme kann es zu Hautirritationen, blauen Flecken oder leichten Schmerzen an der Einstichstelle kommen. Die Impfungen sind für alle Teilnehmer kostenlos.

2 Studienziele

2.1 Primäre Studienziele

Im Rahmen dieser Studie soll die impfinduzierte humorale und zelluläre Immunantwort gegen Tetanus und Diphtherie bei Personen unterschiedlicher Altersgruppen untersucht werden. Bei diesen medizinisch indizierten Impfungen, die nicht aufgrund dieser Studie, sondern im Rahmen von Routine-Auffrischungen stattfinden, soll durch Anamnese, sowie durch drei Blutentnahmen geklärt werden,

- ob ältere Personen nach 5 Jahren in gleicher Weise gegen Tetanus und Diphtherie geschützt sind, wie junge Erwachsene, bei denen die letzte Impfung 10 Jahre zurückliegt.

- ob Auffrischungsimpfungen nach 5 Jahren bei älteren Personen die gleiche Immunantwort induzieren wie bei jungen Erwachsenen nach 10 Jahren.

2.2 Sekundäre Studienziele

Außerdem soll geklärt werden, welche biologischen Parameter (Leber- und Nierenfunktion, Hormone, Entzündungsparameter, Parameter des Fettstoffwechsels, Parameter, die mit der Zellalterung assoziiert sind) mit dem Impferfolg korrelieren und als prädiktive Marker für den Impferfolg geeignet sind.

3 Studienbeschreibung

3.1 Studiendesign

Es handelt sich um eine unkontrollierte, offene Phase IV-Studie

3.2 Primärer Endpunkt der Studie

Quantifizierung der impfinduzierten Immunantwort 28 Tage nach der Impfung

3.3 Probandenzahl

Im Rahmen dieser Studie sollen 100 junge Erwachsene (25-40 Jahre; m/w 1:1) und 100 ältere Personen (über 60 Jahre; m/w 1:1) untersucht werden. In die Studie werden Probanden aufgenommen, die gemäß den offiziellen Impfempfehlungen Auffrischungsimpfungen erhalten. Entsprechend werden dies junge Erwachsene sein, die 10 Jahre nach der letzten Auffrischung eine Impfung gegen Tetanus, Diphtherie, Pertussis und Polio erhalten. Die Gruppe der älteren Probanden besteht aus Personen, die vor 5 Jahren gegen Tetanus, Diphtherie, Pertussis und Polio geimpft wurden und bei denen deshalb jetzt eine Impfung gegen Tetanus, Diphtherie und Pertussis indiziert ist (siehe 1.1).

3.4 Studiendesign/Methodik

Rekrutierung der Probanden

An der Landessanitätsdirektion werden regelmäßig ältere Personen geimpft. Personen, die vor 5 Jahren eine Impfung gegen Tetanus/Diphtherie/Pertussis/Polio erhalten haben, erhalten schriftlich eine Erinnerung, dass eine Auffrischung gegen Tetanus/Diphtherie/Pertussis indiziert ist. Gleichzeitig werden sie eingeladen an unserer Studie teilzunehmen und sich insgesamt 3 Blutentnahmen (insgesamt 95ml) zu unterziehen (Text siehe Anlage). Die Rekrutierung junger Probanden erfolgt durch Zeitungsannoncen (Text siehe Anlage) und Aushänge an öffentlichen Plätzen (z.B. Universität). Dabei werden Personen, bei denen die letzte Impfung 10 Jahre zurückliegt und deshalb eine Auffrischung medizinisch indiziert ist, eingeladen an unserer Studie teilzunehmen. Diese Probanden erhalten eine Auffrischungsimpfung gegen Tetanus/Diphtherie/Pertussis und Polio, die sie auch ohne Teilnahme an der Studie erhalten würden, und unterziehen sich insgesamt 3 Blutentnahmen (insgesamt 95ml. Als Gegenleistung für die Teilnahme an der Studie erhalten die Probanden die entsprechende Impfung kostenlos.

Blutentnahmen/Impfungen/Anamnese

Im Rahmen der Studie werden drei Blutentnahmen durchgeführt. Bei der ersten Blutentnahme (unmittelbar vor der Impfung) werden 35ml venöses Blut abgenommen. 5ml Blut werden zur Serumgewinnung verwendet und aus 30ml (Na-Heparin) werden periphere Blutlymphozyten (PBL) isoliert. Bei der zweiten Blutentnahme (7 Tage nach der Impfung) werden 5ml Blut zur Serumgewinnung verwendet und aus 30ml (Li-Heparin) werden periphere Blutlymphozyten (PBL) isoliert. Bei der dritten Blutentnahme (28 Tage nach der Impfung) werden 15ml Blut zur Serumgewinnung sowie 10ml für die Erstellung eines Blutbilds und zur Analyse der Gerinnung abgenommen. Insgesamt ergibt sich damit eine Blutentnahme von 95ml.

Geimpft wird mit zugelassenen Kombinationsimpfstoffen. Für ältere Probanden ist diese ein Impfstoff gegen Tetanus/Diphtherie/Pertussis (Boostrix, GlaxoSmithKline) bzw. für junge Probanden ein Impfstoff gegen Tetanus/Diphtherie/Pertussis/Polio (Boostrix Polio, GlaxoSmithKline) gemäß der Empfehlung des Obersten Sanitätsrats (vgl. 1.1).

Mittels eines Fragebogens werden bekannte, bestehende Erkrankungen und Lebensumstände wie z.B. Nikotin- und Alkoholkonsum, sportliche Aktivität etc. erfasst.

Read-out Parameter und Methoden

Analysen aus dem Serum:

Es werden die spezifischen Antikörperkonzentrationen gegen Tetanus und Diphtherie vor bzw. nach der Impfung mittels ELISA-Assays bestimmt.

Außerdem werden biologische Parameter bestimmt und mögliche Korrelationen mit dem Impferfolg untersucht. Ziel dieser Analyse ist die Identifizierung von prädiktiven Markern für den Impferfolg. Dabei werden folgende Parameter erfasst:

- Leber- und Nierenfunktion

Um schwere Leber- und Nierenfunktionsstörungen, die Einfluss auf andere erfasste Parameter haben könnten auszuschließen werden Bilirubin, GGT, GOT, GPT sowie Elektrolyte und Kreatinin in einem Routinelabor bestimmt.

- Hormone

Eine Reihe von Studien verweisen auf einen Zusammenhang zwischen Hormonen und dem Alterungsprozess. Aus diesem Grund werden in einem Routine-Labor die Konzentrationen von Testosteron, FSH, FT3, FT4, Östradiol, Progesteron und Prolactin bestimmt. Außerdem wird der PSA-Wert (Prostata-spezifisches Antigen) mittels ELISA bestimmt.

- Entzündungsparameter

Bei älteren Personen kommt es zu subklinischen Entzündungsprozessen und verschiedene Entzündungsparameter wurden in der Literatur mit der Immunfunktion, "Frailty" und dem Mortalitätsrisiko bei älteren Personen in Verbindung gebracht. Im Rahmen dieser Studie werden deshalb die Konzentrationen von IL-6, IL-10, C-reaktivem Protein und Neopterin, sowie die IDO-Aktivität (errechnet aus den Konzentrationen von Tryptophan und Kynurenin) bestimmt.

- Fettstoffwechsel

Bioaktive Substanzen, die von Fettzellen produziert werden (Adipokine) spielen im Energiestoffwechsel eine entscheidende Rolle. Erniedrigte Konzentrationen von Leptin und Adiponectin wurden mit erhöhter Mortalität in sehr alten Personen in Verbindung gebracht. Im Rahmen dieser Studie werden deshalb die Konzentrationen dieser beiden Adipokine bestimmt.

- Latente CMV-Infektion

Eine Reihe von Studien zeigt, dass eine latente Infektion mit dem Zytomegalovirus (CMV) Alterungsprozesse des Immunsystems beeinflusst und verstärkt. Auch ein Einfluss auf den Impferfolg wurde diskutiert. Mittels ELISA wird deshalb die Konzentration CMV-spezifischer Antikörper bestimmt.

- Parameter, die mit der Zellalterung assoziiert sind

In verschiedenen Tiermodellen wurde gezeigt, dass Mutationen innerhalb des IGF (insuline-like growth factor)-Signalweges die Lebensspanne verändern können. Diesem Signalweg wird deshalb eine zentrale Rolle bei der Zellalterung zugeschrieben. Im Rahmen dieser Studie sollen die Konzentrationen von IGF, IGFBP-3 (IGF-binding protein) und IGFBP-6 mittels ELISA untersucht werden. Auch die Konzentrationen von dkk-3 (Dickkopf-3) und TL1A (TNF superfamily ligand 1A), Proteinen, die an unserem Institut an seneszenten Epithel- bzw. Endothelzellen untersucht werden, werden mittels ELISA bestimmt.

Analysen aus Vollblut

Aus dem Vollblut wird ein Differential-Blutbild erstellt und die Gerinnung bestimmt. Diese Analysen werden von einem zertifizierten Routine-Labor durchgeführt.

Analysen aus isolierten PBL:

Die zelluläre Immunantwort gegen Tetanus und Diphtherie wird mittels ELISPOT-Assays analysiert. Für diesen Assay werden CD4+ T-Zellen durch Tetanus- bzw. Diphtherietoxoid stimuliert und die Produktion von Zytokinen (Interferon-gamma, Interleukin 5 und Interleukin 10) auf Einzelzellniveau untersucht. Außerdem werden einzelne Antikörper-produzierende B-Zellen mittels B-Zell-ELISPOT detektiert.

Zusätzlich werden bei allen Probanden mittels FACS-Analyse Oberflächenmoleküle auf Immunzellen analysiert. Dabei werden die Proteine CD3, CD4, CD5, CD8, CD14, CD16, CD19, CD20, CD22, CD25, CD27, CD28, CD38, CD45RA, CD62L, CD138 und FoxP3 quantitativ erfasst. Mit diesen Markern können einzelnen Zellpopulationen (T-Zellen, B-Zellen, Monozyten, NK-Zellen), sowie Subpopulationen innerhalb der T- und B-Zellen (naive, memory, Effektor-Zellen, Plasmazellen, Plasmablasten) unterschieden werden.

Außerdem wird die Telomerlänge von PBL mittels in situ Hybridisierung (fluoreszente PNA-Sonde) und FACS-Analyse bestimmt. Die Telomerlänge dient als Maß für die bisherige Proliferation von Zellen, da bei jeder Zellteilung die Telomerlänge abnimmt. Es wurde gezeigt, dass ausdifferenzierte, seneszente T-Zellen kurze Telomere aufweisen und dass die Telomerlänge von humanen PBL mit einer erhöhten Mortalität assoziiert ist.

3.5 Zeitplan

Die Studiendauer ist insgesamt auf 1 Jahr festgelegt. Beginn der Studie ist im Mai 2009.

4 Probandenpopulation

4.1 Einschlusskriterien

An der Studie können gesunde Personen teilnehmen, die nach dem Aufklärungsgespräch die Einverständniserklärung unterzeichnen.

Gruppe 1: Personen im Alter von 25-40 Jahren, bei denen die letze Tetanus/Diphtherie-Impfung 10 Jahre zurückliegt

Gruppe 2: Personen über 60 Jahre, bei denen die letzte Tetanus/Diphtherie-Impfung 5 Jahre zurückliegt.

4.2 Ausschlusskriterien

Personen, die an einer akuten Erkrankung (z.B. Erkältung, Grippe etc.), einer chronisch entzündlichen Erkrankung (z.B: Morbus Chron, Multiple Sklerose etc.) oder einer chronischen Hepatitis (HBV oder HCV) leiden, sowie HIV-positive Personen können an dieser Studie nicht teilnehmen. Ausgeschlossen sind außerdem Transplantatempfänger, Personen, die sich schon einmal einer Chemotherapie unterzogen haben und Personen unter immunsuppressiver Therapie. Schwangere Frauen können gemäß AMG nicht an der Studie teilnehmen. Bei gebärfähigen Frauen (Gruppe 1: 25-40 Jahre) wird vor dem Einschluss in die Studie von einem Arzt ein Schwangerschaftstest durchgeführt um eine Schwangerschaft auszuschließen.

5 Prüfmedikation

5.1 Beschreibung der Prüfmedikation

Boostrix:

Boostrix (GlaxoSmithKline AG) ist zur Auffrischungsimpfung gegen Tetanus, Diphtherie und Pertussis bei Personen ab dem vollendeten 4. Lebensjahr indiziert. Der Impfstoff wird für diese Studie als Einzeldosen bei der Apotheke zum Tiroler Adler (Innsbruck) bezogen. Eine Einzeldosis enthält 0,5ml Suspensionslösung in einer Fertigspritze. Die Impfantigene (Tetanustoxoid, Diphtherietoxoid und Pertussisantigene) sind an Aluminiumhydroxid und Aluminiumphosphat adorbiert. Daneben entält der Impfstoff Formaldehyd, 2-Phenoxyethanol, Polysorbat 80, Natriumchlorid, Glycin und Wasser für Injektionszwecke. Die Impfstoffe werden bei 2-8°C gelagert. Die Haltbarkeit der Impfstoffe ist auf den Einzelpackungen angegeben.

Boostrix Polio:

Boostrix Polio(GlaxoSmithKline AG) ist zur Auffrischungsimpfung gegen Tetanus, Diphtherie, Pertussis und Polio bei Personen ab dem vollendeten 4. Lebensjahr indiziert. Der Impfstoff wird für diese Studie als Einzeldosen bei der Apotheke zum Tiroler Adler (Innsbruck) bezogen. Eine Einzeldosis enthält 0,5ml Suspensionslösung in einer Fertigspritze. Die Impfantigene (Tetanustoxoid, Diphtherietoxoid, Pertussisantigene und inaktivierte Polioviren) sind an Aluminiumhydroxid und Aluminiumphosphat adorbiert. Daneben enthält der Impfstoff Medium 199, Neomycin, Polymyxin, Polysorbat 80, Natriumchlorid und Wasser für Injektionszwecke. Die Impfstoffe werden bei 2-8°C gelagert. Die Haltbarkeit der Impfstoffe ist auf den Einzelpackungen angegeben.

Beide Impfstoffe dürfen nicht verabreicht werden bei bekannter Überempfindlichkeit, die bei früherer Verabreichung des entsprechenden Impfstoffs aufgetreten ist, oder gegen einen der Bestandteile des Impfstoffes.

5.2 Verzeichnis der Neben- und Wechselwirkungen

Die Fachinformationen der Impfstoffe enthalten eine detaillierte Aufstellung der Neben- und Wechselwirkungen. Im folgenden sind häufige und sehr häufige Nebenwirkungen aufgeführt. Gelegentliche, seltene und sehr seltene Nebenwirkungen sind in der Fachinformation aufgelistet.

Boostrix:

Bei Erwachsenen kann es zu Schmerzen, Rötungen und Schwellung an der Einstichstelle kommen. Außerdem kann es zu Kopfschmerzen, Unwohlsein, Erbrechen, Müdigkeit, Benommenheit oder Fieber kommen.

Boostrix Polio:

Bei Erwachsenen kann es zu Schmerzen, Rötungen und Schwellung an der Einstichstelle kommen. Außerdem kann es zu Müdigkeit, Fieber, Unwohlsein, Kopfschmerzen, Bauchschmerzen, Übelkeit und Erbrechen kommen.

5.3 Behandlungsschema

5.3.1 Anweisungen für die Applikation

Es erfolgt eine einmalige Impfung mit einer Dosis des entsprechenden Impfstoffs.

5.3.2 Notfallmaßnahmen

Die Impfung wird von Ärzten durchgeführt, die große Erfahrung mit Impfungen haben. Die entsprechende Ausrüstung für notfallmedizinische Betreuung ist vorhanden.

5.3.3 Begleitmedikation

Die Probanden können regelmäßig eingenommene Medikamente während der Studie weiter einnehmen.

5.4 Lagerung, Aus- und Rückgabe sowie Dokumentation der Prüfmedikation (Drug Accountability)

Die Impfstoffe werden von der Apotheke zum Tiroler Adler bezogen. Die Impfstoffe werden am Institut für Biomedizinische Alternsforschung gelagert und nach Bedarf an die Landessanitätsdirektion, wo die Impfungen durchgeführt werden geliefert. Die Zahl der gekauften und verimpften Dosen wird dokumentiert. Nach dem Verfallsdatum dürfen die Impfstoffe nicht mehr verwendet werden. Die Lagerung erfolgt bei 2-8°C. Die Temperatur wird im Prüfordner

dokumentiert. Die Verwendung der Impfstoffe wird im Drug Accountability Log im Prüfordner dokumentiert

6 Studienablauf

6.1 Termin 1 (Tag 0)

Jeder Proband muss vor Aufnahme in die Studie informiert werden. Die Aufklärung erfolgt persönlich durch den behandelnden Arzt und schriftlich durch die Probandeninformation. Erst nach Klärung aller Fragen des Probanden wird dieser gebeten, zwei Exemplare der Einwilligungserklärung zu unterschreiben und eigenhändig zu datieren. Dem Probanden wird anschließend ein Exemplar der Probandeninformation/Einwilligungserklärung ausgehändigt; das zweite Exemplar wird im Prüfordner aufbewahrt.

Es erfolgt die Erfassung der demografischen Daten (Personendatenblatt) und die Überprüfung der Ein- und Ausschlusskriterien (Fragebogen Ein-und Ausschlusskriterien). Danach wird eine Blutentnahme durchgeführt (35ml) und die Impfung verabreicht (Boostrix für ältere Probanden, Boostrix Polio für junge Probanden).

6.2 Termin 2 (Tag 7)

Beim zweiten Termin wird der Fragebogen zu Grunderkrankungen und Lebensstil (Sport, Rauchen, Alkohol) ausgefüllt. Außerdem werden 35ml Blut abgenommen. Lokale Beschwerden an der Einstichstelle der Blutentnahme und Nebenwirkungen der Impfung (Lokalreaktion, systemische Beschwerden) werden abgefragt und erfasst.

6.3 Termin 3 (Tag 28)

Beim dritten Termin werden 25ml Blut abgenommen. Lokale Beschwerden an der Einstichstelle der Blutentnahme (Termin 2) werden abgefragt und erfasst. Außerdem wird nochmals nach Nebenwirkungen der Impfung, die seit dem letzten Termin aufgetreten sind gefragt und diese erfasst.

6.3.1 Laboruntersuchungen

Die beim 1. Termin (Tag0) an der Landessanitätsdirektion gewonnen Blutproben werden täglich zum Institut für Biomedizinische Alternsforschung transportiert. Dort erfolgt die Bestimmung von Antikörpertitern gegen Tetanus und Diphtherie. Die zelluläre Immunantwort gegen Tetanus und Diphtherie wird mittels ELISPOT bestimmt. Außerdem werden verschiedene Lymphozyten-Populationen quantifiziert (FACS-Analyse).

Die 2. Blutentnahme (Tag7) erfolgt am Institut für Biomedizinische Alternsforschung, so dass ein Transport nicht notwendig ist. Aus diesen Proben werden wiederum die Antikörpertitern gegen Tetanus und Diphtherie bestimmt. Die zelluläre Immunantwort gegen Tetanus und Diphtherie wird mittels ELISPOT bestimmt. Außerdem werden verschiedene Lymphozyten-Populationen quantifiziert (FACS-Analyse).

Diese Messungen werden sowohl vor also auch nach der Impfung durchgeführt, da es sich um Auffrischungsimpfungen handelt. Die Probanden besitzen also bereits vor der Impfung in geringem

Ausmaß Antikörper und reaktive Immunzellen gegen einzelne Impfantigene. Diese Immunantwort wird durch die Impfung verstärkt. In dieser Studie soll dieser sogenannte "Booster-Effekt" untersucht werden. In Vorversuchen konnte gezeigt werden, dass spezifische T-Zellen und Antikörper-produzierende B-Zellen 7 Tage nach der Impfung im peripheren Blut nachweisbar sind. Die Zahl dieser Zellen in der Peripherie sinkt nach diesem Zeitpunkt wieder ab. Deshalb ist für die Analyse der zellulären Immunantwort eine Blutentnahme 7 Tage nach der Impfung notwendig.

Die 3. Blutentnahme (Tag28) erfolgt ebenfalls am Institut für Biomedizinische Alternsforschung. Ein Teil des Materials wird täglich durch das Labor Dr. Philadelphy abgeholt, wo Routine-Laborparameter (Bilirubin, GGT, GOT, GPT ,Elektrolyte, Kreatinin, Testosteron, FSH, FT3, FT4, Östradiol, Progesteron,Prolactin, CRP, IGF) analysiert werden. Außerdem wird ein Differential-Blutbild erstellt und die Gerinnung bestimmt.

Am Institut für Biomedizinische Alternsforschung werden außerdem der PSA-Wert (Prostata-spezifisches Antigen), Serum-Konzentrationen von IGFBP-3 (IGF-binding protein) und IGFBP-6, dkk-3 (Dickkopf-3) und TL1A (TNF superfamily ligand 1A), Leptin, Adiponectin IL-6, IL-10 und Neopterin, sowie die IDO-Aktivität (errechnet aus den Konzentrationen von Tryptophan und Kynurenin) bestimmt. Die Konzentration von Zytomegalovirus (CMV)- spezifischen Antikörpern wird bestimmt.

Der Anstieg der Antikörper-Titer gegen Tetanus und Diphtherie nach Impfung erfolgt langsamer als die T-Zell-Antwort. Maximale Antikörper-Titer werden 28 Tage nach der Impfung erreicht. Deshalb werden zu diesem Zeitpunkt nochmals die Tetanus- und Diphtheriespezifischen Antikörpertiter bestimmt. Dieser Zeitpunkt ermöglicht es auch unsere Daten mit anderen Studien zu vergleichen, da nahezu alle Studien, die sich mit Antikörper-Titern nach Impfungen beschäftigen, diesen Zeitpunkt für die Analyse verwenden.

6.4 Ausscheiden von Probanden (Drop-out)

Probanden können jederzeit auf eigenen Wunsch vorzeitig und ohne Angabe von Gründen aus der Studie ausscheiden. Außerdem können Probanden durch den Prüfer aus Gründen der Gesundheitsgefährdung aus der Studie ausgeschlossen werden. Ein Ausscheiden aus der Studie hat keinen negativen Folgen für die zukünftige Behandlung der Probanden.

6.5 Vorzeitiger Abbruch der klinischen Prüfung

Hierfür wurden keine Kriterien festgelegt, da ein vorzeitiger Abbruch der Studie nicht vorgesehen ist.

7 Dokumentation von Nebenwirkungen

Beim 2. und 3. Termin werden die Probanden nach lokalen und systemischen Reaktionen nach der Impfung befragt und diese werden im CRF dokumentiert. Außerdem werden die Probanden nach Beschwerden an der Einstichstelle der Blutentnahme gefragt und dies ebenfalls im CRF dokumentiert.

Schwere und unerwartete Nebenwirkungen werden vom Prüfer gemäß den gesetzlichen Bestimmungen an die Ethikkommission und das BASG gemeldet.

8 Dokumentation

Es liegt in der Verantwortung des Prüfers, dass die Durchführung der Prüfung in Übereinstimmung mit den GCP-Richtlinien, dem AMG sowie dem Prüfplan erfolgt und dass die Daten korrekt in den CRF eingetragen werden. Alle in dieser Studie erhobenen Daten müssen von entsprechend autorisierten Personen in den CRF eingetragen werden. Dies gilt auch für Daten von Probanden, die aus der Prüfung ausgeschlossen wurden.

Der Prüfer vermerkt die Teilnahme auf einer speziellen Probandenidentifizierungsliste. Sie dient der Möglichkeit der späteren Identifikation der Probanden und enthält die Probandennummer, den vollständigen Namen des Probanden, sein Geburtsdatum und das Datum der Aufnahme in die klinische Prüfung.

8.1 Erhebungsbogen (CRF)

Sämtliche Probandendaten und Untersuchungsergebnisse sollen in die speziell für diese Studie erstellten CRFs (Case Report Forms) eingetragen werden.

Die Erhebungsbögen dürfen nur mit einem **schwarzen Kugelschreiber** ausgefüllt werden. Korrekturen sind so vorzunehmen, dass die alte Eintragung lesbar bleibt (das Verwenden von Korrekturmitteln ist nicht erlaubt). Korrekturen müssen von der autorisierten Person, die diese vornimmt, signiert und datiert werden. Daten, die nicht verfügbar sind oder die nicht erhoben wurden, müssen als solche klar erkenntlich sein (NA oder ND). Die Gründe dafür sollten ggf. dokumentiert werden.

Der Prüfer stellt sicher, dass sämtliche Daten der Probanden unverzüglich, leserlich, vollständig, korrekt und in Übereinstimmung mit den Probandenakten in die CRFs eingetragen werden.

Die ausgefüllten Originalseiten werden durch den Monitor hinsichtlich Plausibilität und Vollständigkeit überprüft.

8.2 Prüfordner

Im Prüfordner bewahrt der Prüfer diejenigen Dokumente auf, die für die klinische Prüfung erforderlich sind. Im Rahmen des Monitorings wird der Prüfordner auf Aktualität und Vollständigkeit entsprechend den Regularien überprüft. Nach Abschluss oder Abbruch der Studie ist der Prüfordner 15 Jahre lang aufzubewahren.

8.3 Aufbewahrung der Daten

8.3.1 Aufbewahrungspflichten des Sponsors

Alle wesentlichen Unterlagen der klinischen Prüfung müssen vom Sponsor für die Dauer von 10 Jahren nach Ende oder Abbruch der klinischen Prüfung aufbewahrt werden. Der Sponsor muss die prüfungsrelevanten Unterlagen und Dokumente entsprechend den gesetzlichen Bestimmungen archivieren.

8.3.2 Aufbewahrungspflichten des Prüfers

Aufzeichnungen und Dokumente, die im Zusammenhang mit der Prüfung oder der Vergabe von Prüfmedikation stehen (z. B. Erhebungsbögen, Einwilligungserklärungen, Listen der Medikationsverteilung und andere relevante Unterlagen) müssen von den Prüfern mindestens 10 Jahre aufbewahrt werden.

Die Krankenblätter und andere Originaldaten müssen über den längst möglichen Zeitraum, den das Krankenhaus, die Institution oder die private Praxis gestattet, aufbewahrt werden.

9 Monitoring

9.1 Monitoring

Der Prüfer erklärt sich bereit, dass der Monitor Datenüberprüfungen vornimmt, um die zufriedenstellende Erhebung der Daten und die Einhaltung des Prüfplanes zu sichern.

Weiterhin erklärt sich der Prüfer zur Zusammenarbeit mit dem Monitor bereit und dazu, ihm, wann immer nötig, alle erforderlichen Informationen zukommen zu lassen. Dies beinhaltet den Zugang zu allen Unterlagen, die mit der Studie im Zusammenhang stehen. Dem Monitor wird außerdem ermöglicht, die Datenüberprüfung sowie den Vergleich mit den relevanten Probandenakten gemäß den SOPs (z. B. des KKS) und den ICH-GCP-Richtlinien in den vorher festgelegten Zeitabständen durchzuführen, um die Einhaltung des Prüfplans und die kontinuierliche Aufzeichnung der Daten zu gewährleisten. Dabei werden alle medizinischen Originalbefunde, die als Quelle für die Informationen im CRF bzw. in der Datenbank notwendig sind, überprüft. Der Proband hat sich durch Unterzeichnung der Einwilligungserklärung mit einer solchen Überprüfung einverstanden erklärt.

Weitere Aufgaben des Monitors sind:

- Prüfung, ob das Prüfzentrum den Anforderungen der klinischen Prüfung entspricht (Probandenpopulation, Geräte, Aufbewahrung des Studienmaterials usw.)
- Überprüfung des Prüfordners auf Vollständigkeit und Aktualität
- Originaldatenabgleich
- Überprüfung der vorschriftsmäßigen Meldung von SAEs
- Überprüfung der ordnungsgemäßen und sicheren Lagerung sowie der Haltbarkeit der Prüfmedikation
- Zählen der zurückgegebenen Prüfmedikation (Drug accountability)

Der Monitor ist verpflichtet, alle Informationen vertraulich zu behandeln und den grundsätzlichen Anspruch der Probanden auf Integrität und Schutz ihrer Privatsphäre zu wahren.

10 Dateneingabe und Datenmanagement

Der Probanden-Fragebogen / Case Report Form enthält Informationen über akute und chronische Erkrankungen des Probanden. Weiters sind Informationen über Allergien, den Impfstatus und gegenwärtig eingenommene Medikamente des Probanden verzeichnet. Außerdem werden Daten der Blutentnahmen, des Aufklärungsgesprächs, sowie die Ergebnisse der durchgeführten Untersuchungen dokumentiert. Auf dem Fragebogen / Case Report Form sind keine persönlichen Daten (Name, Geburtsdatum etc.) des Probanden sondern nur ein zugeteilter Probandencode vermerkt. Eine Zuordnung des Codes zu den persönlichen Daten erfolgt auf dem

Probandendatenblatt, das vom Institut für Biomedizinische Alternsforschung gesondert archiviert und vertraulich behandelt wird. Für alle Auswertungen, sonstigen Aufzeichnungen und Publikationen werden nur die Probandencodes verwendet.

Die Prüfbögen, die Originale der Probandeninformation und der Einwilligungserklärung werden in der Probandenkartei des Instituts für biomedizinische Alternsforschung archiviert. Die Daten werden durch das Institut für Biomedizinische Alternsforschung mit Hilfe der Software Excel und SPSS verwaltet und bearbeitet.

11 Statistik

11.1 Fallzahlplanung

Diese explorative Studie hat zum Ziel, Prädiktoren für einen Impferfolg für Tetanus bzw. Diphterie zu identifizieren. Potenzielle Prädiktoren sind die Altersgruppe (25-40 Jahre vs. 60-100 Jahre) und verschiedene biologische Serumparameter.

Die Fallzahlschätzung basiert auf einer explorativen multiplen logistischen Regression mit Impferfolg als abhängiger Variable und Alter und den Serumparametern als unabhängigen Variablen.

Basierend auf einer angenommenen Zahl von 10 effektiven Prädiktoren (Alter, Geschlecht und 8 Serumparameter), einer durchschnittlichen Impferfolgs¬wahrscheinlichkeit von 50% und einem Verhältnis von jüngeren und älteren Probanden von 1:1 ergibt sich nach auf der Fallzahlschätzungsregel von Harrell (ca. 10 benötigte Ereignisse pro identifiziertem Prädiktor) eine Gesamtfallzahl von 200 Probanden, d.h. 100 Probanden pro Altersgruppe.

11.2 Statistische Methoden

Zielgrößen

Die primäre Zielgröße der Analysen ist die Impferfolgswahrscheinlichkeit.

Datenanalyse

Es erfolgt eine explorative Datenanalyse mittels multipler logistischer Regression. Die Variablenselektion erfolgt in zwei Schritten. Zunächst werden potenzielle Prädiktoren mittels einfacher logistischer Regression (univariat) vorselektiert ($p<0.20$). Aus diesen Kandidatenvariablen wird anschließend in einer multiplen logistischen Regression mittels Forward-Selektionsverfahren (Einschlusskriterium $p<0.05$) das finale Prädiktionsmodell bestimmt. Es werden Effektmaße (Odds Ratios) mit 95%-Konfidenzintervallen geschätzt und berichtet. Interaktionsterme zwischen Alter und Serumparametern und zwischen Serumparametern werden explorativ untersucht. Aus dem Gesamtmodell wird ein prädiktiver Score (linearer Prädiktor der logistischen Regression) zur Schätzung der Impferfolgswahrscheinlichkeit abgeleitet. Der prädiktive Wert des Gesamtmodells wird als c-Wert angegeben.

Referenz: (Harrell FE. Regression Modeling Strategies with Applications to Linear Models, Logistic Regression and Survival Analysis. Springer Series in Statistics. Springer-Verlag, New York, 2001).

12 Berichterstattung

12.1 Prüfbericht

Sämtliche Informationen, die diese Studie betreffen, sind vertraulich zu behandeln. Die statistische Auswertung und die Erstellung eines integrierten Abschlussberichtes werden vom Institut für Biomedizinische Alternsforschung (IBA) in Zusammenarbeit mit dem Institut für Public Health, Medical Decision Making und HTA realisiert und von allen weiteren verantwortlichen Personen evaluiert und unterzeichnet. Alle in diesem Bericht enthaltenen Informationen sind streng vertraulich.

12.2 Publikation

Die Ergebnisse dieser Studie werden veröffentlicht. Die Veröffentlichung oder ein Vortrag der Ergebnisse bedürfen einer vorhergehenden Kommentierung und Genehmigung durch die Kooperationspartner. Für alle Veröffentlichungen gilt, dass der Datenschutz für alle Probandendaten gewahrt bleibt.

13 Ethische, rechtliche und administrative Aspekte

13.1 Verantwortlichkeiten von Sponsor und Prüfer

Der Sponsor der klinischen Prüfung übernimmt gemäß AMG die Verantwortung für die Veranlassung, Organisation und Finanzierung der durchzuführenden klinischen Prüfung. Sponsor und Prüfer stellen dabei sicher, dass die klinische Prüfung im Einklang mit den bestehenden Gesetzen und Bestimmungen, entsprechend den ICH-GCP-Richtlinien (1996), der Deklaration von Helsinki (1996) sowie den Vorschriften des AMG und der GCP-Verordnung (2004) durchgeführt wird. Der Prüfer akzeptiert die Anforderungen des unterzeichneten Prüfplans.

Verantwortlichkeiten des Prüfers sind u. a.:

- Verständnis der Eigenschaften, der in der Fachinformation beschriebenen Prüfmedikation
- Verständnis und Umsetzung des Behandlungsplans
- Sicherstellung, dass ausreichend Zeit und Kapazität für die Durchführung der Prüfung zur Verfügung stehen
- Korrekte Sammlung und Dokumentation der Daten, Berichterstattung
- Bereitstellung sämtlicher Daten für den Sponsor, Monitor oder entsprechende Behörden für Audits und/oder Inspektionen
- Sicherstellung, dass die Informationen über Probanden sowie alle vom Sponsor erhaltenen Informationen von allen in die Prüfung involvierten Personen vertraulich behandelt werden.

13.2 Votum der Ethikkommission und Behördenmeldung

Die Antragstellung erfolgt bei der Ethikkommission der Medizinischen Universität Innsbruck und beim Bundesamt für Sicherheit im Gesundheitswesen im Auftrag des Sponsors durch Prof. Beatrix Grubeck-Loebenstein.

13.3 Probandeninformation und Einwilligungserklärung

Vor Beginn der Prüfung muss jeder Proband dem Prüfer schriftlich seine Einwilligung erklären, nachdem er zuvor in mündlicher und schriftlicher Form vollständig in für ihn verständlicher Weise über Wesen, Bedeutung und Tragweite der klinischen Prüfung aufgeklärt worden ist. Der Inhalt dieser Aufklärungsinformation wird auf der Einwilligungserklärung dokumentiert.

Die Einwilligungserklärung des Probanden zur Teilnahme an der klinischen Prüfung erfolgt mit Datum und Unterschrift des Probanden sowie des Arztes. Dem Probanden wird ein Exemplar der unterschriebenen Probandeninformation/Einwilligungserklärung ausgehändigt. Das zweite Exemplar legt der Arzt im Prüfordner ab.

Es wird ausdrücklich darauf hingewiesen, dass bis zum Vorliegen einer rechtsgültigen Einwilligungserklärung des Probanden keinerlei Untersuchungen im Zusammenhang mit der Studie durchgeführt werden dürfen.

13.4 Probandenversicherung

Im Auftrag des Sponsors wurde die vorgeschriebene Probandenversicherung gemäß AMG für alle Probanden bei folgendem Versicherer abgeschlossen:

Name des Unternehmens:	HDI Versicherung
Versicherungsnummer:	1375586
Anschrift:	Edelsinnstr. 7-11, 1120 Wien
Telefon:	0509-05 501 420
Telefax:	0509-05 501 420

Damit sind studienbedingte Gesundheitsschädigungen mit einer maximalen Deckungssumme von 500.000 Euro pro Teilnehmer versichert. Diese Versicherung deckt alle eventuellen Schädigungen, die der Proband direkt oder indirekt durch die Prüfmedikation oder Eingriffe im Zusammenhang mit der klinischen Prüfung erleidet, ab.

Der Proband kann die Versicherungsbedingungen beim Prüfer einsehen und auf Wunsch in Kopie erhalten.

Der Proband wird darauf aufmerksam gemacht, dass für ihn kein Versicherungsschutz besteht für einen Unfall, der ihm auf dem Weg zur und von der Teilnahme an der Studie zustößt.

13.5 Datenschutz und Schweigepflicht

Die Erhebung, Weitergabe, Speicherung und Auswertung persönlicher Daten innerhalb dieser Studie erfolgt nach gesetzlichen Bestimmungen (Sächsisches Datenschutzgesetz, Bundesdatenschutzgesetz). Voraussetzung dafür ist die freiwillige Zustimmung der Probanden im Rahmen der Einwilligungserklärung vor Teilnahme an der klinischen Prüfung. Die Probanden werden dazu innerhalb der Aufklärung über diese klinische Prüfung über Folgendes informiert:

1. Im Rahmen dieser klinischen Prüfung erhobene Daten werden auf Papiererhebungsbögen oder elektronischen Datenträgern aufgezeichnet, streng vertraulich behandelt und ohne Namensnennung (pseudonymisiert) nur weitergegeben an

   – den Auftraggeber der Studie zur wissenschaftlichen Auswertung und Bewertung von unerwünschten Ereignissen,
   – die zuständigen Bundesbehörde, die Ethikkommission der Medizinischen Universität Innsbruck und die europäische Datenbank zur Überprüfung der ordnungsgemäßen Durchführung der Studie sowie zur Bewertung von Studienergebnissen und unerwünschten Ereignissen.

2. Soweit dies für die Überprüfung der klinischen Prüfung notwendig ist, können autorisierte und zur Verschwiegenheit verpflichtete Beauftragte des Auftraggebers (Monitore, Auditoren) und/oder der zuständigen Überwachungsbehörde in die beim Prüfer vorhandenen personenbezogenen Daten Einsicht nehmen. Für diese Maßnahme wird der Prüfer von der ärztlichen Schweigepflicht entbunden.

3. Die Einwilligung zur Erhebung und Verarbeitung der personenbezogenen Daten im Rahmen dieser klinischen Prüfung ist unwiderruflich. Ein Proband wird darüber aufgeklärt, dass er jederzeit die Teilnahme an der klinischen Prüfung – ohne Angabe von Gründen und ohne irgendwelche folgenden Nachteile – beenden kann. Im Falle des Widerrufs der Einwilligungserklärung werden die bis zu diesem Zeitpunkt gespeicherten Daten ohne Namensnennung weiterhin verwendet, soweit dies erforderlich ist, um Wirkungen des zu prüfenden Arzneimittels festzustellen und zu sichern, dass schutzwürdige Interessen der betroffenen Person nicht beeinträchtigt werden.
